# Integrative analysis of mRNA and miRNA expression profiles and somatic variants in oxysterol signaling in early‐stage luminal breast cancer

**DOI:** 10.1002/1878-0261.13495

**Published:** 2023-08-18

**Authors:** Petr Holý, Veronika Brynychová, Karolína Šeborová, Vojtěch Haničinec, Renata Koževnikovová, Markéta Trnková, David Vrána, Jiří Gatěk, Kateřina Kopečková, Marcela Mrhalová, Pavel Souček

**Affiliations:** ^1^ Third Faculty of Medicine Charles University Prague Czech Republic; ^2^ Biomedical Center, Faculty of Medicine in Pilsen Charles University Pilsen Czech Republic; ^3^ Toxicogenomics Unit National Institute of Public Health Prague Czech Republic; ^4^ Department of Oncosurgery MEDICON Prague Czech Republic; ^5^ Aeskulab, k.s. Prague Czech Republic; ^6^ Comprehensive Cancer Center Novy Jicin Hospital Novy Jicin Czech Republic; ^7^ Department of Surgery EUC Hospital Zlin and Tomas Bata University in Zlin Czech Republic; ^8^ Department of Oncology, Second Faculty of Medicine Charles University and Motol University Hospital Prague Czech Republic; ^9^ Department of Pathology, Second Faculty of Medicine Charles University and Motol University Hospital Prague Czech Republic

**Keywords:** breast cancer, integrative analysis, interaction network, multiomics, oxysterols, survival

## Abstract

Oxysterols, oxidized derivatives of cholesterol, act in breast cancer (BC) as selective estrogen receptor modulators and affect cholesterol homeostasis, drug transport, nuclear and cell receptors, and other signaling proteins. Using data from three highly overlapping sets of patients (*N* = 162 in total) with early‐stage estrogen‐receptor‐positive luminal BC—high‐coverage targeted DNA sequencing (113 genes), mRNA sequencing, and full micro‐RNA (miRNA) transcriptome microarrays—we describe complex oxysterol‐related interaction (correlation) networks, with validation in public datasets (*n* = 538) and 11 databases. The *ESR1‐CH25H‐INSIG1‐ABCA9* axis was the most prominent, interconnected through miR‐125b‐5p, miR‐99a‐5p, miR‐100‐5p, miR‐143‐3p, miR‐199b‐5p, miR‐376a‐3p, and miR‐376c‐3p. Mutations in *SC5D*, *CYP46A1*, and its functionally linked gene set were associated with multiple differentially expressed oxysterol‐related genes. *STARD5* was upregulated in patients with positive lymph node status. High expression of hsa‐miR‐19b‐3p was weakly associated with poor survival. This is the first study of oxysterol‐related genes in BC that combines DNA, mRNA, and miRNA multiomics with detailed clinical data. Future studies should provide links between intratumoral oxysterol signaling depicted here, circulating oxysterol levels, and therapy outcomes, enabling eventual clinical exploitation of present findings.

## Graphical abstract

Oxysterols affect multiple key processes in cells, including cancer. Here, for the first time, we combine data on oxysterol‐related gene expression, genetic variation, and expression of regulating micro‐RNA molecules, to gain insight into oxysterol‐mediated signaling in breast cancer. Most importantly, we uncover multiple potential regulatory interactions, especially between the estrogen receptor (*ESR1*), *CH25H*, *INSIG1*, and *ABCA9*.

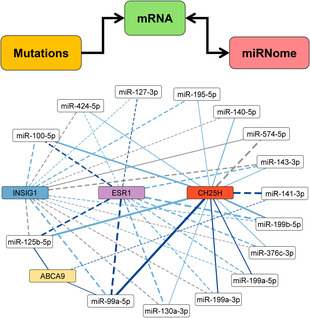

**Abbreviations**
25‐HC: 25‐hydroxycholesterol
3D: three‐dimensional
BC: breast cancer
CI: confidence interval
DFS: disease‐free survival
DIABLO: Data Integration Analysis for Biomarker discovery using Latent variable approaches for Omics studies
DNA‐seq: high‐throughput DNA sequencing
ERα: estrogen receptor alpha
FDR: false discovery rate
GATK: Genome Analysis Toolkit
HUGO: Human Genome Organisation
miRNA: micro‐RNA
miRNA‐seq: high‐throughput miRNA sequencing
mRNA‐seq: high‐throughput mRNA sequencing
OS: overall survival
PCA: principal component analysis
pN: pathological node status
pT: pathological tumor size
RPM: reads per million
SERM: selective estrogen receptor modulator
TCGA: The Cancer Genome Atlas
TCGA‐GA: TCGA dataset obtained using the Genome Analyzer platform
TCGA‐HS: TCGA dataset obtained using the HiSeq platform
TPM: transcripts per million

## 1 Introduction

Breast cancer (BC) is the most frequent cancer diagnosis worldwide for females (25% of cases), and in 2020, it surpassed lung cancer as the most common cancer overall. Despite advances in diagnosis and treatment, BC remains the number one cancer by mortality in women globally (15.5% of all cancer deaths in females in 2020) [1]. Resistance of cancer cells to both conventional and targeted treatment represents an ongoing problem for successful therapy. With the advent of modern genomic methods, precision medicine, promising effective treatment individualized for every patient, moves ever closer to clinical practice [2]. However, studies in precision oncology in patients often focus on only one type of data, for example, genomics, transcriptomics, epigenomics, proteomics, or other omics‐type datasets, without combining multiple types of data for the same patients, missing an opportunity for deeper insight [3].

Oxysterols are a group of cholesterol derivatives, generated endogenously either enzymatically or by autoxidation, or being introduced through the diet [4, 5]. A growing body of evidence points to oxysterols playing significant roles in the regulation of multiple vital cellular pathways and in a wide range of pathologies. In BC in particular, oxysterols have been shown to function as selective estrogen receptor modulators (SERM) [6]. In addition, more general effects of various oxysterols on cholesterol homeostasis, drug transport, nuclear and cell receptors, and other key signaling proteins mean that oxysterols have been implicated in a number of cancer types [7] and can also affect the efficacy of anticancer therapy [8].

Micro‐RNAs (miRNAs) are short (20–24 nucleotides) noncoding nucleic acids canonically regulating gene expression by modulation of mRNA cleavage or repression of translation [9], which have also been shown to have both tumor‐suppressing and tumor‐promoting properties [10]. Interestingly, they are often expressed in clusters, offering a chance to more easily detect and target miRNAs in cancer research and treatment [11]. Expression of many important lipid metabolism and homeostasis genes/proteins is regulated by miRNAs [12, 13]. Some of those genes/proteins, like the liver X receptor, *ABCA1* or *ABCG2*, and many others, are known to be also modulated by oxysterols [14].

Recently, we showed that somatic mutations in *CYP46A1* and functionally related genes, as well as in a group of genes associated with progesterone receptor status, are associated with poor survival in early estrogen‐receptor (ER)‐positive BC patients of the luminal subtype [15]. However, for deeper insight, findings in the genetic area should be supplemented with their gene expression context. In this study, we have therefore combined our existing somatic variation data from targeted DNA sequencing (DNA‐seq) of a panel of 113 oxysterol‐related genes (Table S1, [15]) with respective mRNA expression data obtained by mRNA sequencing (mRNA‐seq) and with the complete miRNA transcriptome data obtained by microarrays, from the same BC patients. The genes and their roles in oxysterol signaling are summarized in our previous publications [15, 16, 17].

The aim of this study was to document a potential mRNA‐miRNA interaction network of oxysterol signaling in BC and to complement this with a range of analyses of mRNA and miRNA data together with DNA mutation and clinical data. The hypotheses generated would then inform future focused experimental studies in the underexplored area of oxysterol signaling in BC.

## 2 Materials and methods

### 2.1 Patients

A total of 162 incident BC female patients, diagnosed in the Department of Oncosurgery Medicon and Motol University Hospital, both in Prague, and EUC Hospital in Zlin, Czech Republic, throughout 2001–2013, were included in the study. For their full clinical characteristics, see Table S2.

Diagnosis of all patients was confirmed histologically according to standard diagnostic procedures [18]. Immunohistochemical evaluation of hormone receptor expression was based on a 1% cutoff. ERBB2 (erb‐b2 receptor tyrosine kinase 2; also known as HER2) status was tested by immunohistochemistry (IHC); 3+ scores were considered positive, and 0 and 1+ were considered negative. In the case of 2+ scores, fluorescent *in situ* hybridization was used for status confirmation. The threshold between high and low expression of proliferative marker Ki‐67 was 14% according to Cheang et al. [19]. Molecular subtypes were classified based on IHC, following published recommendations [20, 21]. Exclusion criteria for the study were the refusal of informed consent of the patient, preoperative chemotherapy or endocrine therapy, stage IIIB and higher, subtype other than luminal, and lack of histological diagnosis. Disease‐free survival (DFS) was defined as the time between surgery and the first disease relapse, including local relapses and death, or the last control in remission. Overall survival (OS) was calculated as the time from surgery to death or the last follow‐up date. The logistics of sample collection, storage, and processing have been described elsewhere [17].

Procedures performed in the present study followed the 1964 Helsinki Declaration and its later amendments or comparable ethical standards. The Ethical Commission of the National Institute of Public Health in Prague approved the study protocol (approvals no. 9799‐4, NT13679, and NT14055‐3). All patients were informed about the study, and only those who agreed and signed the informed consent of the patient further participated in the study.

### 2.2 Total RNA extraction, quantification, and quality control

Each tumor tissue sample was pulverized by mortar and pestle under liquid nitrogen, and total RNA was isolated using the TRIZOL reagent (ThermoFisher Scientific, Waltham, MA, USA) following the manufacturer's protocol and kept at −80 °C. RNA was quantified using the Quant‐iT RiboGreen RNA Assay Kit (Invitrogen, Carlsbad, OR, USA) on the plate reader Infinite M200 (Tecan Group Ltd., Männedorf, Switzerland). Quality (RNA integrity number—RIN) and quantity of RNA were assessed on the Bioanalyzer 2100 instrument using the RNA 6000 Nano kit (both Agilent Technologies Inc., CA, USA).

### 2.3 miRNA microarrays

In total, 125 samples were included. The miRNA Microarray System with miRNA Complete Labeling and Hyb Kit and the SurePrint G3 Unrestricted miRNA 8x60K v19.0 microarray slides (both Agilent Technologies Inc., Santa Clara, CA, USA) was used according to the manufacturer's protocol. Briefly, 100 ng of total RNA per sample was dephosphorylated, labeled with Cyanine 3‐pCp, hybridized, washed, and scanned using the Agilent SureScan Microarray Scanner instrument with *scan control* v9 software, and data were extracted using the *feature extraction* software v11.5 (both Agilent Technologies Inc.).

For initial quality control and filtering, the *genespring* v14.9 software was used (Agilent Technologies Inc.). First, values were quantile normalized and log_2_ transformed. Samples shown to be outliers by 3D PCA analysis were excluded. Then, to eliminate low expression values that could be biased by background, miRNA entities were filtered so that their signal intensity values were between the 20–100th percentile. To eliminate rarely expressed miRNAs, only probe sets that were detected in at least 25% of samples were retained. To adjust for batch effects caused by sourcing samples from three different hospitals and measurement by different laboratory operators, the *ComBat* empirical Bayesian algorithm [22, 23] of the *sva* v3.40 *R* package was used with the nonparametric setting (non‐normality of data confirmed by the Shapiro–Wilk test).

### 2.4 mRNA sequencing

In total, 67 samples were available for sequencing. Libraries were prepared from 500 ng of total RNA using the QuantSeq 3′mRNA‐Seq Library Prep FWD for Illumina kit (Lexogen, Vienna, Austria) according to the manufacturer's protocol. Samples with RIN > 3.5 (*n* = 58) were processed by the standard protocol, while samples with RIN < 3.5 (*n* = 9) were processed using the low‐quality RNA protocol. The quality of prepared libraries was checked by Bioanalyzer 2100 using the High Sensitivity DNA kit (Agilent Technologies Inc.), and quantity was measured by qPCR using the KAPA Library Quantification Kit for Illumina® Platforms (Roche, Switzerland) and by the Qubit instrument using the Qubit DNA HS Assay Kit (both ThermoFisher Scientific). The equimolar pool of libraries was sequenced on the NextSeq 500 platform (Illumina Inc., CA, USA) using the High Output kit (1 × 75 bp setting) and targeting 5–6 million reads per sample.

Quality control of sequencing data was performed using *fastqc* v11.9 [24]. For annotation, reference transcriptome gencode v35 [25] (GRCh38.p13) was used. Quantification of protein‐coding transcripts via pseudoalignment was done using *kallisto* v0.48 [26] with default settings. For correlation analyses, the count data were normalized to transcripts per million (TPM) and were log_2_ scaled. For the purposes of the study, only data pertaining to the 113 oxysterol‐related genes were used.

### 2.5 DNA sequencing

Sequencing data of tumor and nontumor DNA originated from our previous study and are described there [15]. In brief, DNA short mutation data were obtained via high‐throughput panel sequencing of 113 oxysterol‐related genes (Table S1) using the SureSelect XT Low Input platform with target enrichment by a custom‐designed probe set (0.8 Mb; all Agilent Technologies Inc.). Libraries were sequenced on the NextSeq platform in 150 bp paired‐end mode (Illumina Inc.). Analysis of somatic variants utilized a matched normal sample for every tumor sample, with the pair sequenced simultaneously. Bioinformatic analysis to obtain a list of variants used primarily the *genome analysis toolkit* 4.1.9 (*GATK*, Broad Institute of MIT and Harvard, Cambridge, MA, USA) and followed the GATK Best Practices [27]. *annovar* v2020‐06‐08 and the RefSeq database [28] were used for annotation.

### 2.6 Integrative bioinformatic analysis, statistics, and visualization

For all statistical testing, *r* v4.1 or v4.2 [29] was used. The normality test (Shapiro–Wilk) of both miRNA and mRNA data showed non‐normal distribution (median *P*‐values across miRNAs and mRNAs 0.009 and 0.006, respectively); therefore, the nonparametric Spearman rank correlation test was chosen for its robustness in comparison with the Pearson method when correlating non‐normal data. For differential expression analysis of mRNAs, *edger* v3.36.0 [30, 31] was used with default settings. For survival analyses, the Kaplan–Meier method with the log‐rank test was performed by *survival* v3.3 and Cox regression by *spss* v16.0 (SPSS, Inc., Chicago, IL, USA). For the correction of *P*‐values for multiple testing, the Benjamini–Hochberg false discovery rate (FDR) method was used [32]. *P*‐values < 0.05 after FDR correction were considered statistically significant. Reported *P*‐values are unadjusted, unless stated otherwise.

To evaluate the validity of mRNA‐miRNA correlated pairs, we used the *r* package *multimir* v1.14 (database v2.3.0) [33] to query eight databases of predicted and three databases of experimentally validated mRNA‐miRNA interactions (Table S3). Only the top 20% (by default) of interactions by interaction score were considered.

For network visualization, *cytoscape* v3.10 [34] was used. The *pheatmap* v1.0.12 [35] package was used for creating heatmaps. For analysis, evaluation, and visualization of integrated multiomic signatures, we employed the *mixomics* v6.22.0 package [36], using principal component analysis (PCA) and multiblock sparse partial least squares—discriminant analysis (also called DIABLO) methods [36, 37].

### 2.7 Validation datasets

For validation purposes, we used one mRNA and two miRNA expression datasets originating from The Cancer Genome Atlas (TCGA) Breast Cancer project (BRCA) (https://www.cancer.gov/tcga) and downloaded via the UCSC Xena platform (https://xenabrowser.net/datapages/; accessed 25 March 2022).To increase similarity with the primary cohort, the datasets were reduced to tumor samples of only female patients with ER‐positive tumors, disease stage I or II, and without neoadjuvant treatment (*n* = 538 for mRNA dataset; *n* = 157 for the first miRNA dataset, TCGA‐GA; *n* = 374 for the second miRNA dataset, TCGA‐HS). The mRNA dataset contained all patients from the two miRNA datasets.

The original mRNA dataset (20 530 mRNAs, Illumina HiSeq 2000, log_2_(norm_count+1) normalized values) was subsetted to the 113 oxysterol‐related genes ([15], Table S1), and gene names were updated to adhere to the latest Human Genome Organisation (HUGO) nomenclature [38]. The TCGA‐GA miRNA dataset [log_2_(RPM + 1) normalized values] was obtained using the Illumina Genome Analyzer sequencing platform. The TCGA‐HS miRNA dataset [log_2_(RPM + 1) normalized values] was prepared using the Illumina HiSeq 2000 sequencing platform. Due to differences in the methodology used, we decided to treat these as two different cohorts and analyze them separately. TCGA‐GA and TCGA‐HS were filtered so that only those miRNAs that were analyzed in the primary cohort (see Section 3) were retained.

MiRNA accession numbers were converted to/from their mature miRNA names (miRBase v19) using *mirbaseconverter* v1.18 [39].

## 3 Results

### 3.1 Patients

**Fig. 1:**
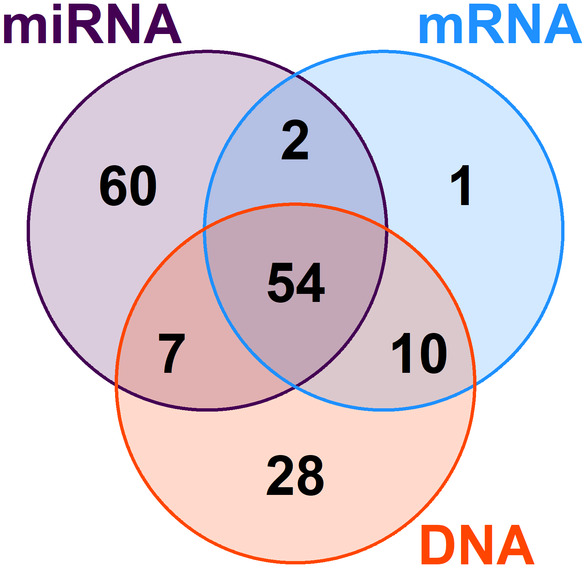
Venn diagram of all breast tumor samples included in the study, divided by type of data obtained. DNA—short somatic variant data of oxysterol‐related gene panel by targeted DNA‐seq. miRNA—expression data for full miRNA transcriptome by microarray. mRNA—expression data for oxysterol‐related gene panel by mRNA‐seq.

Out of 125 patients with their miRNAs measured, two were deemed technical outliers due to assay overload and were excluded from further analyses. All mRNA‐seq data (*n* = 67) passed quality controls. The overlap between the two groups was 56 patients. For a Venn diagram of the DNA‐seq, mRNA‐seq, miRNA microarray, and overlapping subcohorts (162 patients in total, clinical data in Table S2), see Fig. 1.

### 3.2 Correlation analyses

#### 3.2.1 Co‐expression of miRNAs

In order to reveal which co‐expressed miRNAs or their clusters are prominent in our cohort, and to confirm whether the data are representative of larger, more heterogeneous cohorts, we performed miRNA‐miRNA correlation analyses in both the study cohort and two TCGA validation cohorts and compared the results.

**Fig. 2:**
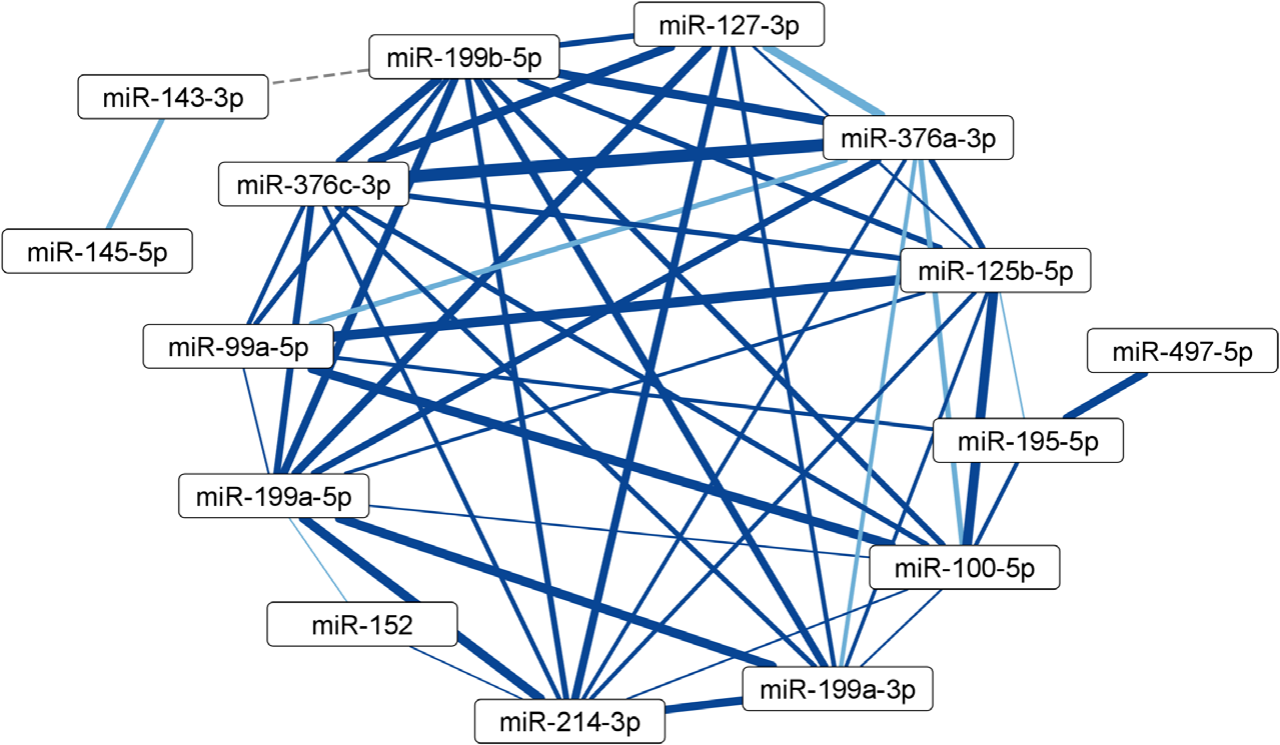
Largest group of co‐expressed miRNAs, confirmed by validation in TCGA. Line thickness corresponds to strength of correlation (*r* between 0.80 and 0.99). Confirmation by validation datasets: both (dark blue), one (light blue), none (gray dashed line). ‘hsa’‐omitted from miRNA names for brevity. For other groups, see Figs S1 and S2.

From the initial 2027 miRNA entities detected in 125 tumor samples, 280 miRNAs and 123 samples passed all our filters. The miRNA expression data were then correlated across all 123 samples with each other to reveal co‐expressed (or mutually exclusive) miRNAs. For network analysis, we selected only strong interactions with a correlation coefficient (*r*) ≤ −0.8 or ≥ 0.8. No negative correlation could be classified as strong (*r* ≤ −0.8). However, we identified 230 strong positive interactions, all of which passed the significance threshold (adj. *P* < 0.05) after FDR (Table S4). We repeated the data processing and analysis with two separate filtered (see Section 2.7) miRNA‐seq TCGA‐BRCA datasets: TCGA‐GA (*n* = 157) and TCGA‐HS (*n* = 374). Out of 230 total unique statistically significant miRNA‐miRNA interactions found in the original dataset, 79 were also found in both TCGA‐GA and TCGA‐HS, three only in TCGA‐GA, and 14 only in TCGA‐HS. For the majority of interactions, the positive correlation coefficient dropped below the minimum of 0.80 of the primary cohort (median 0.55, Table S4). One hundred and thirty‐four interactions were confirmed in neither of the validation datasets. Notably, whether an interaction was confirmed or not was highly group‐specific. The second largest group (15 miRNAs, Fig. 2), three groups of five miRNAs, one group of three miRNAs, and 15 miRNA pairs were confirmed completely or almost completely, while one group of eight nodes had one out of 11 interactions confirmed (all in Fig. S1). Several other groups (2–41 miRNAs) were not confirmed at all (Fig. S2). For the complete list of interactions in the study and validation datasets, including correlation coefficients and FDR‐adjusted *P*‐values, see Table S4.

To see whether co‐expression is affected by patient characteristics relevant in hormone signaling, we repeated the correlation analyses in the primary cohort separated into clinical subgroups based on menopausal status, PR status, and intrinsic tumor subtype. The interactions with the largest differences in correlation coefficients are listed in Table S5. None of the miRNA pairs belonged to significantly correlated groups of the full cohort (Fig. 2, Figs S1 and S2). In addition, many individual correlations were not statistically significant, making these results hard to interpret.

#### 3.2.2 Co‐expression of oxysterol‐related mRNAs

To inform our subsequent mRNA‐miRNA analyses, we correlated mRNA expression values of all 113 oxysterol‐related genes (Table S1) across all 67 samples to see any potentially co‐expressed genes. No correlation could be considered strong with adjusted *P*‐value ≤0.05 (*r* between −0.42 and 0.74. Hierarchical clustering analysis did not reveal any strong clusters, although there were multiple positively and negatively correlated gene groups (Fig. S3). To see how representative our dataset was of larger, more heterogeneous cohorts, we performed validation in the TCGA data (*n* = 538). Out of 869 significant correlations, 418 agreed (direction of correlation the same) with TCGA, 130 disagreed and 321 were not significant in TCGA (Table S6).

Analogously to the previous section, we compared correlations between clinical subgroups based on menopause status, PR status and intrinsic tumor subtype (Table S7). These comparisons suffer from similar limitations as the previous miRNA‐miRNA results, making interpretation problematic.

#### 3.2.3 Network analysis of oxysterol‐related mRNA and miRNA expression

**Fig. 3:**
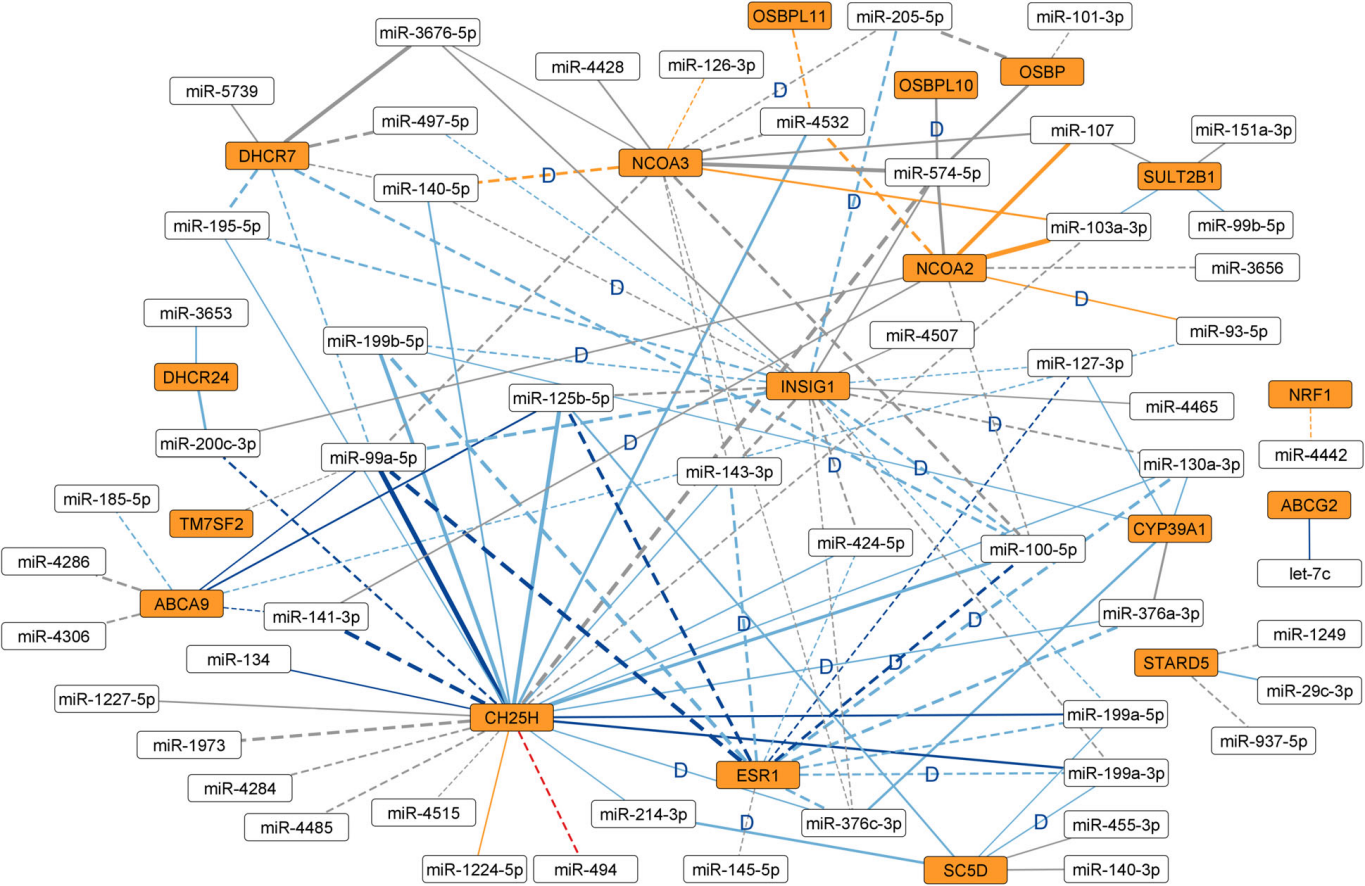
Interaction network based on mRNA‐miRNA expression correlations significant after FDR adjustment. mRNAs in orange and miRNAs in white. Negative correlation in dashed lines and positive in solid lines. Dark blue lines—validated by both TCGA datasets; light blue lines—validated by one TCGA dataset; gray lines—not validated by either TCGA dataset; orange lines—disputed by one TCGA dataset; red lines—disputed by both TCGA datasets. ‘D’ in the middle of a line—interaction found in at least one of 11 databases queried (listed in Table 1; for methodology, see chapter 2.6 and Table S3). ‘hsa‐’ omitted from miRNA names for brevity.

Finally, we performed correlation analysis between the mRNA (113 oxysterol‐related genes, Table S1) and miRNA data (see Section 2.7) across 56 overlapping samples of the primary cohort. For the 123 interactions that reached significance (network in Fig. 3, listed in Table S8), we queried databases of *in silico* predicted or experimentally validated mRNA‐miRNA interactions by *multiMiR* and found 14 (11.4%) and nine (7.3%) interactions to be either predicted or validated, respectively (Table 1). Four interactions, that is, *INSIG1* with hsa‐miR‐130a‐3p, *ESR1* with hsa‐miR‐130a‐3p and with hsa‐miR‐145‐5p, and *NCOA2* with hsa‐miR‐200c‐3p were in databases of both predicted and validated interactions.

**Table 1:** Significant mRNA‐miRNA interactions that were also found in databases, with validation by TCGA data.

mRNA	miRNA (hsa‐miR‐…)	Correlation coefficient (*r*)	FDR adj. *P*‐value	Databases of predicted^a^	Databases of validated^a^	TCGA‐GA (*r*; FDR adj. *P*‐value)	TCGA‐HS (*r*; FDR adj. *P*‐value)
*CH25H*	376a‐3p	0.50	0.036	miRanda		NS	0.24; 5.87E‐05
*CH25H*	376c‐3p	0.49	0.038	miRanda		NS	0.34; 1.63E‐09
*ESR1*	100‐5p	−0.57	0.006		mirTarBase	−0.30; 8.41E‐03	−0.25; 2.49E‐05
*ESR1*	130a‐3p	−0.58	0.004	DIANA‐microT EIMMo miRDB PicTar PITA	mirTarBase	NS	−0.19; 1.90E‐03
*ESR1*	143‐3p	−0.56	0.007	miRanda		NS	−0.22; 2.69E‐04
*ESR1*	145‐5p	−0.49	0.042	EIMMo	TarBase	NS	NS
*ESR1*	199a‐3p	−0.53	0.018	PITA		NS	−0.15; 0.022
*INSIG1*	100‐5p	−0.52	0.021		mirTarBase	NS	NS
*INSIG1*	130a‐3p	−0.52	0.021	DIANA‐microT EIMMo miRanda miRDB PITA TargetScan	TarBase	NS	NS
*INSIG1*	140‐5p	−0.50	0.037	DIANA‐microT miRDB		NS	NS
*INSIG1*	205‐5p	−0.55	0.009		TarBase	NS	−0.18; 4.01E‐03
*INSIG1*	424‐5p	−0.52	0.021		TarBase	NS	NS
*NCOA2*	141‐3p	0.50	0.031		TarBase	NS	NS
*NCOA2*	200c‐3p	0.50	0.031	DIANA‐microT EIMMo miRanda miRDB	miRTarBase	NS	NS
*NCOA2*	93‐5p	0.50	0.033	PITA		NS	−0.21; 5.36E‐04
*NCOA3*	140‐5p	−0.57	0.006	DIANA‐microT MicroCosm		NS	0.13; 0.045
*NCOA3*	205‐5p	−0.50	0.031	DIANA‐microT		NS	NS
*OSBPL10*	574‐5p	0.55	0.012	PITA		NS	NS
*SC5D*	199a‐3p	0.49	0.040	miRanda		NS	0.22; 1.72E‐04

^a^ For individual database versions and respective links, see Table S3.

For further validation, we correlated the TCGA mRNA data with the filtered (see Section 2.7) TCGA‐GA and the TCGA‐HS data, separately, and restricted the results to only those significant after FDR correction. Out of the 123 original interactions, 14 were found to agree (direction of correlation the same) with both validation datasets and 46 with only one of the datasets while not being present in the other. One interaction disagreed (hsa‐miR‐494 with *CH25H*, direction of correlation opposite) with both datasets, 10 with only one of the datasets while not being present in the other. Fifty‐two interactions were not found in either validation dataset (Table S8). In terms of the interactions found in databases, those of *CH25H*, *ESR1*, *INSIG1*, and *SC5D* agreed with at least one TCGA dataset, although the correlations were considerably weaker in all cases. In the case of *NCOA2* and *NCOA3*, the correlation direction was shown to be opposite in one of the TCGA datasets (Table 1, Fig. 3).

We again repeated the analyses to see whether the network would differ between clinical groups (Table S9) based on menopause status, PR status, and intrinsic tumor subtype. Only one prominent mRNA‐miRNA pair from our original mRNA‐miRNA network showed a large difference between subgroups (*CH25H*‐hsa‐miR‐494: 0.17 in luminal A, −0.78 in luminal B patients) (Table S9). More differences were observed than in the case of miRNA‐miRNA and mRNA‐mRNA comparisons (Tables S5 and S7, respectively), but they were again based mostly on statistically nonsignificant correlations.

### 3.3 Effects of mutation status of oxysterol‐related genes and clinical factors on mRNA expression

**Fig. 4:**
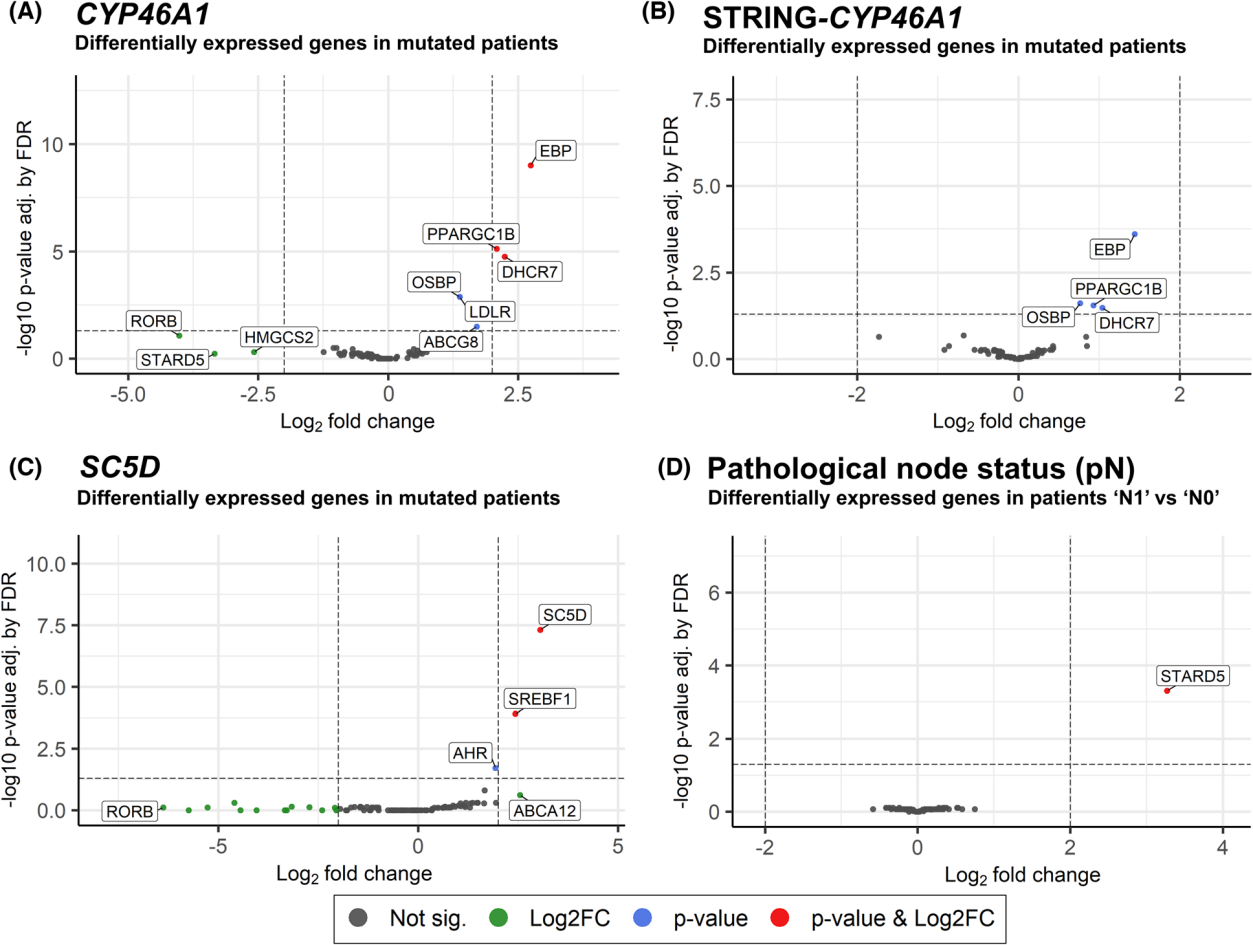
Volcano plots of differentially expressed genes. Patient groups separated by mutation status of (A) CYP46A1, (B) the STRING‐CYP46A1 gene set, and (C) SC5D. (D) Patients separated by their lymph node status (pN; N1 = nodes tumor‐positive, N0 = nodes tumor‐negative). Vertical dashed lines show the threshold for high fold change (log_2_FC = ±2), and horizontal dashed lines show the threshold for significant *P*‐value after FDR (0.05).

We divided patients into groups based on the somatic mutation (any type) status of individual genes or gene sets that were associated with survival or other clinical characteristics in our previous study [15] and/or the mutation status of genes found to be prominent in the mRNA‐miRNA networks. We then performed differential expression analysis between the groups. Patients mutated in *CYP46A1* and in the STRING‐CYP46A1 gene set (nine additional functionally related genes), which were most significantly associated with poor survival in our previous study [15], also showed the largest number of differentially expressed genes. For *CYP46A1*‐mutated patients (*n* = 4), it was *EBP*, *DHCR7*, and *PPARGC1B* that showed not only the largest log_2_ fold change (log_2_FC; 2.74, 2.24, and 2.09, respectively) but also the only significant *P*‐values after FDR (9.71E‐10, 7.47E‐06, and 1.72E‐05, respectively). *OSBP*, *LDLR*, and *ABCG8* were also significant (Fig. 4A). When expanding the cohort to patients mutated in the STRING‐CYP46A1 gene set (*n* = 15), it was *EBP*, *DHCR7*, *PPARGC1B*, and *OSBP* that remained significant (adj. *P*‐values 0.0002, 0.033, 0.028, and 0.024, respectively), but their log_2_FC decreased (1.44, 1.04, 0.93, and 0.76, respectively; Fig. 4B). Patients with mutations in *SC5D* (*n* = 2) saw its mRNA significantly upregulated (log_2_FC = 3.06; adj. *P* = 4.84E‐08; Fig. 4C), along with that of *SREBF1* (log_2_FC = 2.43; adj. *P* = 0.0001), and *AHR* (log_2_FC = 1.93; adj. *P* = 0.019). *SC5D* was also upregulated in patients with *ABCA9* mutations (*n* = 4, log_2_FC = 1.30; adj. *P* = 0.028; Fig. S4). Finally, those mutated in *ESR1* (*n* = 3) had higher expression of *NCOR1* (log_2_FC = 1.71; adj. *P* = 7.85E‐05; Fig. S4).

Finally, we compared expression between groups defined by clinical factors (tumor size and type, node status, molecular subtype, *ERBB2*, and progesterone receptor. Only the lymph node status (pN) of patients led to any differentially expressed genes. Patients with positive regional nodes (pN1; *n* = 28) saw the expression of *STARD5* increase substantially as opposed to those with negative (pN0; *n* = 36) (log_2_FC = 3.26; adj. *P* = 0.0005; Fig. 4D).

### 3.4 Individual associations of mRNA and miRNA expression and clinical factors with survival

We also evaluated potential associations of a range of factors with DFS and OS of patients. First, we compared the survival of 67 patients based on whether they showed the expression of a particular mRNA (*n* = 93) to be high (above or equal to the median) or low (below the median). For especially low‐expressed genes where the median would be zero (*n* = 20), we compared patients with any expression of an mRNA to those without. Two genes showed high expression as being prognostic of poor survival—*LDLR* (OS *P* = 0.003; DFS *P* = 0.020; Fig. S5) and *PPARGC1A* (OS *P* > 0.05; DFS *P* = 0.020; Fig. S6). Similarly, from the list of low‐expressed genes, any expression of *CYP3A4* was associated with worse survival compared with no expression (OS *P* = 0.017; DFS *P* = 0.015; Fig. S7). No results passed the FDR test and none were confirmed in the TCGA cohorts.

**Fig. 5:**
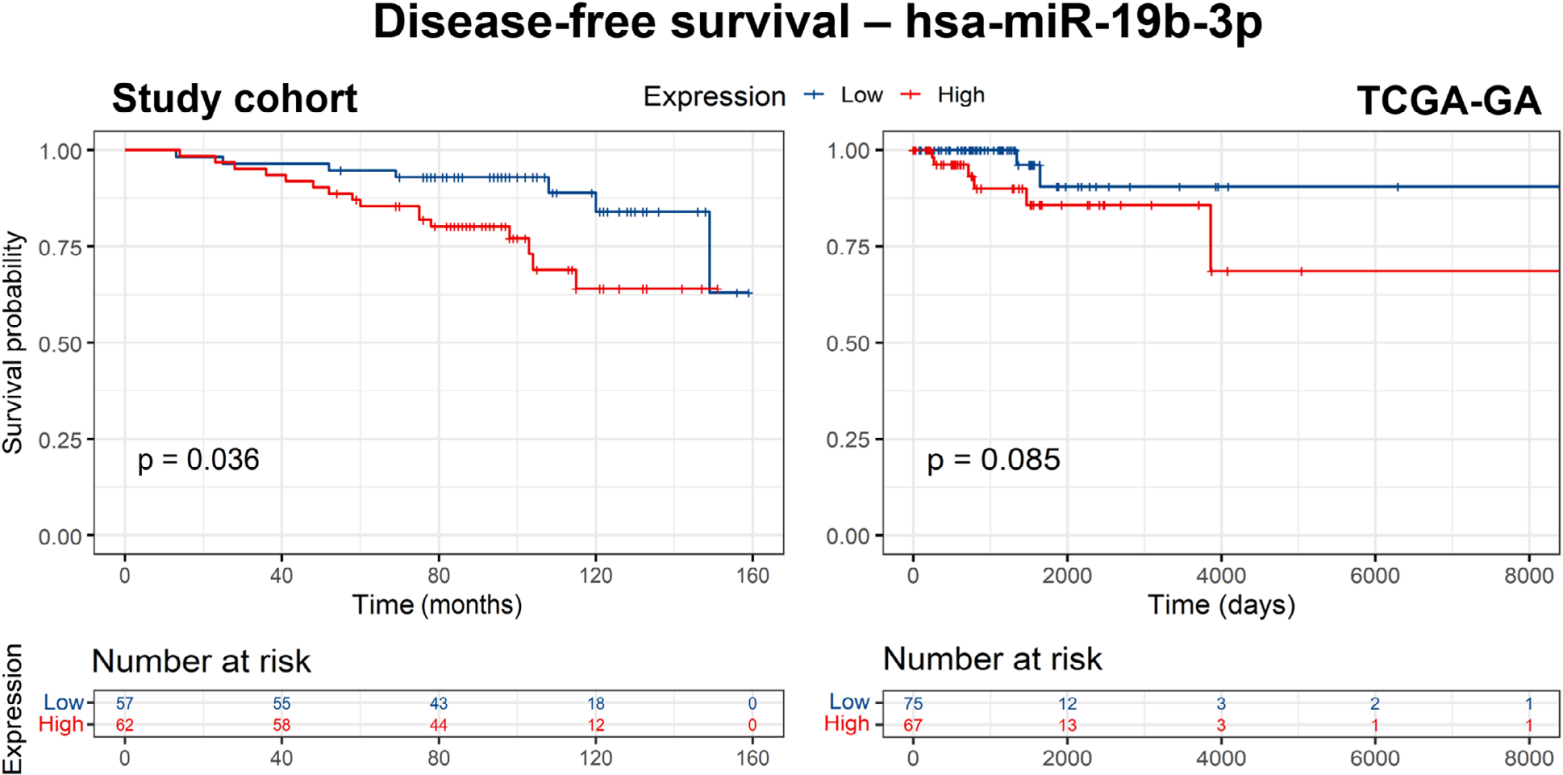
Disease‐free survival of patients in the study cohort and the TCGA‐GA cohort separated by hsa‐miR‐19b‐3p expression level. High = above or equal to median, Low = below median. *P*‐values by log‐rank test, unadjusted.

Analogously, in 123 patients, we compared the survival data of patients based on the expression of a miRNA (*n* = 280, see Sections 2.3 and 3.2). High expression of eight miRNAs was associated either with prolonged (hsa‐miR‐106b‐5p, hsa‐miR‐3653, hsa‐miR‐6069, and hsa‐miR‐6515‐3p), or shortened OS (hsa‐miR‐23b‐3p, hsa‐miR‐4459, hsa‐miR‐4497, and hsa‐miR‐4745‐5p), and that of seven miRNAs was associated with prolonged (hsa‐miR‐222‐3p, hsa‐miR‐1587, hsa‐miR‐4449, hsa‐miR‐4687‐3p, and hsa‐miR‐6069) or shortened (hsa‐miR‐19b‐3p and hsa‐miR‐4745‐5p) DFS (Table S10), although no results remained significant after FDR correction. Only two miRNAs were associated with both OS and DFS. High expression of hsa‐miR‐6069 was associated with prolonged OS (*P* = 0.009) and DFS (*P* = 0.004; Fig. S8), while that of hsa‐miR‐4745‐5p with poor OS (*P* = 0.027) and DFS (*P* = 0.011; Fig. S9). None of these associations reached significance after FDR correction. However, high expression of hsa‐miR‐19b‐3p, which was associated with poor DFS in our data (*P* = 0.036, Fig. 5), showed a similar trend in the TCGA‐GA data, although not significant (*P* = 0.085, Fig. 5), but not in TCGA‐HS data (not shown). However, it was also associated with poor OS in the study cohort, TCGA‐GA, and TCGA‐HS (*P* = 0.17; *P* = 0.14; *P* = 0.05, respectively; Fig. S10), although statistically significant only in TCGA‐HS. In addition, an association with poor disease‐specific survival in TCGA‐HS was close to significance (*P* = 0.080, Fig. S10).

Last, we tested clinical factors (pathologic stage, grade, tumor size (pT), node status (pN), luminal subtype A vs B, progesterone receptor (PR) status, ERBB2 status, Ki‐67 status, and invasive ductal adenocarcinomas vs others, menopause status). Here, only large (> pT1) tumor size was associated with poor survival (Fig. S11) in all patients with available information (OS: *P* = 0.004 in 157 patients; DFS: *P* = 0.012 in 158 patients). In order to determine whether the association of high hsa‐miR‐19b‐3p expression with poor DFS was a prognostic factor independent of clinical parameters, we fitted the Cox regression model with pT as a covariate. Expression of miR‐19b‐3p above median was independently associated with poor DFS, with relapse risk ratio of 2.622 (95% CI: 1.071–6.423; *P* = 0.035).

### 3.5 Integrated oxysterol‐related mRNA‐miRNA prediction model

We used PCA and plotted the first two principal components in mRNA and miRNA data of our samples and of the TCGA‐GA and TCGA‐HS datasets while stratifying the samples by pathologic stage, pN, pT, and OS status (living vs deceased) and PR status (Table S11 for cohort statistics, Figs S12–S16 for plots). None of these factors seemed to define any obvious clusters of samples in any dataset.

Despite this, we attempted to fit multiomic predictive models at least to the largest mRNA‐miRNA dataset TCGA‐HS using the DIABLO framework [37]. Due to the categorical nature of the mutation data (unsuitable for DIABLO), the small number of mutated patients in the genes of interest, and the incomparability of mutation data from TCGA and from our cohort [15], we utilized only the mRNA and miRNA expression data. We again used stage, OS, pN, pT, and PR status as potentially discriminating factors. However, we were unsuccessful in fitting a reliably predictive model, with weighted balanced error rates higher than 40%. Since the initial model showed inadequate predictive power, we did not proceed with validation in the smaller datasets. In hierarchically clustered heatmaps representing the signatures (Figs S17–S19), a slight enrichment of samples with pT > pT1 can be seen in the second largest cluster (Fig. S18). Similarly, a small cluster enriched in PR‐negative patients seems to have a distinct signature (Fig. S19). For lists of mRNAs and miRNAs representing minimal discriminatory signatures with the best fit and for notes on the methodology, see Table S12.

## 4 Discussion

This study fills a gap in the understanding of the genomic and transcriptomic background of oxysterol signaling in early‐stage BC, where a complex multiomic approach has so far been neglected. We present novel hypothetical mRNA‐miRNA interactions, confirm ones already published in multiple databases in other contexts, and validate the majority of findings using large publicly available datasets. Co‐expressed groups of miRNAs are documented and validated as well. In addition, differential expression patterns of oxysterol‐related genes based on their somatic mutation status, as well as nonsignificant (after correction) associations of oxysterol‐related mRNA and miRNA expression with patient survival are presented. Finally, we demonstrate that oxysterol‐related miRNA‐mRNA interactions are not suitable for the fitting of multiomic models that would be predictive of clinical characteristics.

First, we correlated miRNAs between themselves in order to not only reveal either established or novel biological miRNA clusters, which can have clinical relevance in and of themselves [11, 40], but also further inform the following mRNA‐miRNA network analysis. We confirmed that miRNAs tend to be co‐expressed in groups and that only some of these groups can be found in publicly available data, such as TCGA. This is likely due to differences in miRNome versions between the two projects. This highlights the importance of correct reporting of the miRBase version used in any study pertaining to miRNAs. The most strongly correlated and validated group of miRNAs (Fig. 2) was later found to be the backbone of the mRNA‐miRNA network (discussed below).

In the mRNA‐miRNA network analysis (Fig. 3), the highest connectivity was shown by *CH25H*, with the majority of strongest correlations being positive. This is contrasted by *ESR1*, negatively correlated with a series of miRNAs mostly corresponding to the largest confirmed strongly co‐expressed miRNA group. These two genes are highly significantly (*P* adj. < 0.01) linked through hsa‐miR‐100‐5p, hsa‐miR‐125b‐5p, and hsa‐199b‐5p. Other, less significant (*P* adj. < 0.05) links are then via hsa‐miR‐130a‐3p, hsa‐miR‐143‐3p, hsa‐miR‐199a‐3p, hsa‐miR‐199a‐5p, hsa‐miR‐376a‐3p, and hsa‐miR‐376c‐3p. The majority of these interactions were also found in the validation data and many of them in databases as well. This suggests a possible functional link between *ESR1* and *CH25H*. *ESR1* codes for the estrogen receptor alpha (ERα), whose vital importance for BC needs no explanation [41]. Its association with oxysterols is primarily through 27‐hydroxycholesterol, which can act as a SERM [6]. SERMs such as tamoxifen are routinely used for the treatment of ER‐positive BC. *CH25H* produces cholesterol 25‐hydroxylase, a ubiquitously expressed enzyme known for converting cholesterol to an important signaling oxysterol, 25‐hydroxycholesterol (25‐HC), able to bind to a number of receptors [42]. Remarkably, ERα is one of those modulating growth rate of breast and ovarian cancer cells *in vitro* through activation by 25‐HC [43]. Perhaps the interaction between *ESR1* and *CH25H* involves miRNAs as well. It should be noted that *INSIG1*, whose protein is responsible for the maintenance of cholesterol homeostasis by inhibiting cholesterol production, and is also affected by 25‐HC [44], was negatively correlated with *CH25H* in our study (confirmed not in validation data, but in databases), largely via the same miRNAs. This suggests a possible *ESR1*‐*CH25H*‐*INSIG1* interaction network. High expression of *ESR1* in ER‐positive tumors, coupled with upregulation of *INSIG1* and downregulation of *CH25H* (presumably accompanied by lower cholesterol and 25‐HC production, respectively) could involve regulation by miRNAs. However, it has been shown *in vitro* that ER‐positive cells can actually have their cholesterol metabolism and 25‐HC production enhanced compared with ER‐negative BC cells [45]. Cholesterol metabolism upregulation is also a potential marker of resistance to endocrine therapy and poor prognosis [45]. Radically different study designs and objectives, and the fact that our cohort was 100% ER‐positive and very homogeneous in general, unfortunately make direct comparison of these results impossible. Moreover, *ESR1* and *INSIG1* are significantly negatively correlated with *ABCA9* via hsa‐miR‐99a‐5p and hsa‐miR‐125b‐5p. *ABCA9* is a membrane lipid transporter [46, 47] which is downregulated in BC (ER‐positive in most cases) compared with normal tissues [48, 49].

Hsa‐miR‐99a‐5b, hsa‐miR‐125b‐5p, and hsa‐miR‐100‐5p are part of two well‐defined clusters, miR‐99a/let‐7c/miR‐125b‐2 (MIR99AHG, chromosome 21) and miR‐100/let‐7a/miR‐125b‐1 (MIR100HG, chromosome 11). Low level of miR‐99a/let‐7c/miR‐125b‐2 was associated with shorter OS in patients with the luminal A subtype [50]. We also tested this *post hoc* in our cohort (26 luminal A patients) and found similar trends for both OS and DFS, but not statistically significant due to the low number of patients (data not shown).

Previously, the importance of deregulation of hsa‐miR‐99a‐5p, hsa‐miR‐100‐5p, hsa‐miR‐125b‐5p, hsa‐miR‐143‐5p, and hsa‐miR‐376a‐3p in the development of ductal carcinoma *in situ* (DCIS) from normal breast tissue and/or in the development of invasive breast carcinoma from the DCIS has been described. Interestingly, hsa‐miR‐99a‐5p, hsa‐miR‐125b‐5p, and hsa‐miR‐376a‐3p were specifically downregulated in the luminal B (PAM50) subtype [51]. Moreover, hsa‐miR‐99a‐5p, hsa‐miR‐100‐5p, hsa‐miR‐125b‐5p, hsa‐miR‐130a‐3p, and hsa‐miR‐376c‐3p have been proposed as blood biomarkers in breast carcinoma patients [52, 53]. We propose studying all the abovementioned miRNAs, especially in interactions with *CH25H*, *ESR1*, *INSIG1*, and *ABCA9*, in focused functional studies in luminal BC models and patients.

Because oxysterol signaling is hormone signaling‐related, we repeated all the correlation analyses of miRNA‐miRNA, mRNA‐mRNA, and mRNA‐miRNA separately for clinical subgroups based on menopause status, progesterone receptor status, and intrinsic subtype (Tables S5, S7 and S9) to see whether the networks are affected. One aspect connecting all the analyses is that almost none of the largest differences involved the most prominent interactions from the networks of the full cohorts (*n* = 123 for miRNA‐miRNA, *n* = 67 for mRNA‐mRNA, and *n* = 56 for mRNA‐miRNA). Practically, all the largest differences involved peripheral and often overall weakly expressed miRNAs or mRNAs. One exception was the *CH25H*‐hsa‐miR‐494 correlation, which was negative (*r* = −0.53) in the full cohort, which disagreed with both our TCGA validation cohorts (0.27 and 0.30 for TCGA‐GA and TCGA‐HS, respectively, Table S8, Fig. 3, in red). However, luminal A (*n* = 13) patients showed *r* = 0.17 (adj. *P* = 0.983), while luminal B patients (*n* = 29) had *r* = −0.78 (adj. *P* = 0.003). This might point to luminal A patients being more similar to the TCGA cohorts in this particular interaction than the luminal B patients, which clearly accounted for the negative *r* of the full cohort and the only large discrepancy in the full network versus TCGA data (Fig. 3). Unfortunately, a fully comparable subtype‐based correlation analysis in the validation TCGA data was not possible due to incompatibility of the clinical data with ours (TCGA molecular subtypes are based on RNA‐seq data, while our cohort is characterized by clinical immunohistochemistry). Despite this, we *post hoc* compared the correlations in our validation TCGA‐HS cohort reduced only to samples where RNA‐seq‐based classification was unambiguously luminal A (*n* = 63) or luminal B (*n* = 18). In both groups, the correlation was positive (luminal A: *r* = 0.22, adj. *P* = 0.540; luminal B: *r* = 0.371, adj. *P* = 0.788), but these results were again based on low numbers of patients and were not statistically significant. We consider such comparisons between any two networks, especially derived from low number of samples (and different cohort sizes), to be very challenging to statistically analyze and interpret. Such results should be taken with a high degree of caution.

Following up on our previous study where mutations in *CYP46A1*—coding for an enzyme responsible for converting cholesterol into 24S‐hydroxycholesterol—and functionally related genes were weakly associated with poor survival of BC patients [15], we discovered that these same patients have multiple oxysterol‐related genes upregulated as well, namely *EBP*, *PPARGC1B*, and *DHCR7*. These patients were in fact one of only several groups out of hundreds analyzed in this study that had any genes differentially mutated, and they were the ones with the most substantial dysregulation. This adds oxysterol‐related gene expression to the list of genomic differences of this poorly surviving group of patients, although any causal relationships remain unclear and unlikely, especially due to mutations in *CYP46A1* being intronic. The abovementioned genes and their roles in oxysterol signaling have been discussed by us previously [15, 16].

*STARD5* was the one highly upregulated gene based on clinical characteristics, in lymph node‐positive patients compared with negative. *STARD5* is key for cholesterol homeostasis regulation, especially in liver cells [54]. It is indeed hepatocellular carcinoma where it has been recently proposed as a potential diagnostic and prognostic biomarker, with high expression associated with lower tumor grade and better prognosis [55], indirectly in contrast with our results in BC. In our study, it was also correlated with three miRNA: positively with hsa‐miR‐29c‐3p and negatively with hsa‐miR‐937‐5p and hsa‐miR‐1249. To our knowledge, this is the first link, even if indirect, of these miRNAs to any clinical parameter of BC. The potential importance of *STARD5* and its regulation in BC is only starting to emerge, and we consider it an understudied gene indeed.

As an intriguing secondary result not related to oxysterols, hsa‐miR‐19b‐3p emerged as a miRNA whose above‐median expression was associated with worse survival of patients. An association with DFS was found in our cohort and came close to significance in TCGA‐GA. There was a borderline significant result for OS in the TCGA‐HS cohort, with the trend also present in our cohort and TCGA‐GA, although not significant there. Finally, a nearly significant association with DSS was found in TCGA‐HS. Hsa‐miR‐19b‐3p, together with hsa‐miR‐19a‐5p, forms the miR‐19 family (sharing the same seed sequence) and is transcribed in two paralogous clusters—miR‐17‐92 (chromosome 13q31.3) and miR‐106b‐25 (chromosome 7q21; miRBase v22.1). MiR‐17‐92 cluster is overexpressed in a number of tumor types [56], and the miR‐19 family has been shown to be the main oncogenic activator of this cluster through the repression of the tumor suppressor PTEN and the activation of the AKT–mTOR pathway [57]. High expression of miR‐19b in BC tissues is significantly associated with shorter OS in of BC patients in general [58]; however, specific prognostic roles of miR‐19 in different subtypes of BC have not yet been described.

Oxysterol‐related genes were not suitable for training of multiomic statistical models that would reliably discriminate between patient subgroups. However, despite us having applied this methodology on a much smaller dataset than it is intended for (whole transcriptomes), there were indications that with more samples, models able to predict tumor size or PR status of tumors based on our shortlist of 113 oxysterol‐related mRNAs and 280 miRNAs could perhaps be created. The models would benefit from adding more samples, as well as types of data, for example, proteomic or methylomic. Perhaps an improved multiomic model, able to include categorical data such as gene mutation status or clinical factors, could show better predictive ability. To our knowledge, such a model has not been developed yet. However, even if such a model could be fitted, aside from being of theoretical value to systems biology, its applicability in clinical practice would be questionable.

The main limitation of our study is the low numbers of patients in some comparisons, mainly those based on the mutation status of particular oxysterol‐related genes, since these genes are not frequently mutated in our cohort, or in general. These results should therefore be treated with caution. In addition, the number of patients in integrative analyses is limited by the imperfect overlap of our miRNA microarray, mRNA‐seq, and DNA‐seq cohorts. Next, since the present study used a clinically relatively homogeneous set of early‐stage Czech BC patients of Caucasian ethnicity, the results should not be applied to a more general global population. However, large population studies, which do contain similar specific subpopulations of comparable size, usually lack the detailed analysis and focus that this study provides. We therefore believe in the value of studies in specific populations such as ours. It needs to be stressed that the numerous potential mRNA‐miRNA interactions found are based on correlations and lack experimental verification. Even though some of them were validated in two separate external datasets and multiple databases of *in silico* predicted and/or experimentally validated interactions, they should be treated as hypotheses in need of functional confirmation. In addition, some of the comparisons, especially between subgroups (menopause status, PR status, and intrinsic subtype), are based on low patient numbers.

For eventual utilization in clinical practice, the functions of the *ESR1*‐*CH25H*‐*INSIG1*‐*ABCA9* axis and other minor interactions should be investigated in focused experimental *in vitro* and *in vivo* studies and their on/off status should be connected with real oxysterol levels in patients and their therapy outcomes. First, the most influential miRNAs should be identified and then hormonal therapy should be applied to test its efficacy when the signaling is active/inactive. Then, a series of oxysterols should be introduced to test their effects on the system. In case a link between specific oxysterols and therapy, for example, tamoxifen, is found, it could eventually be used for patient prognostication and therapeutic management.

## 5 Conclusions

The present study provides new insights into the mRNA‐miRNA landscape of oxysterol‐related genes in BC in unprecedented detail, in combination with mutation data. The main result is a complex mRNA‐miRNA interaction network, where we reveal a potential *ESR1*‐*CH25H*‐*INSIG1‐ABCA9* subnetwork involving several co‐expressed miRNAs. We also add to the existing evidence linking overexpression of hsa‐miR‐19b‐3p to worse survival of patients with ER‐positive tumors. These results should be used as the basis of follow‐up experimental studies in the area of oxysterol research in cancer.

## Conflict of interest

The authors declare no conflict of interest.

## Author contributions

PH contributed to conceptualization, methodology, software, validation, formal analysis, investigation, resources, data curation, writing—original draft preparation, writing—review and editing, visualization, project administration, and funding acquisition. VB contributed to investigation, data curation, and writing—review and editing. KŠ contributed to methodology, software, data curation, and writing—review and editing. VH contributed to investigation. RK, MT, DV, JG, KK, and MM contributed to resources, data curation, and writing—review and editing. PS contributed to conceptualization, methodology, resources, formal analysis, data curation, writing—review and editing, supervision, project administration, and funding acquisition.

### Peer review

The peer review history for this article is available at https://www.webofscience.com/api/gateway/wos/peer‐review/10.1002/1878‐0261.13495.

## Supporting information


**Fig. S1.** Additional groups of co‐expressed miRNAs.
**Fig. S2.** Groups of co‐expressed miRNAs that were mostly not confirmed by TCGA validation.
**Fig. S3.** Clustered heatmap of mRNA‐mRNA correlations across all 113 oxysterol‐related genes.
**Fig. S4.** Volcano plots of differentially expressed genes.
**Fig. S5.** Kaplan–Meier plots of survival of patients divided by low or high expression (below or above median) of *LDLR* mRNA.
**Fig. S6.** Kaplan–Meier plots of survival of patients divided by low or high expression (below or above median) of *PPARGC1A* mRNA.
**Fig. S7.** Kaplan–Meier plots of survival of patients divided by zero or nonzero expression (none or any level of expression detected) of *CYP3A4* mRNA.
**Fig. S8.** Kaplan–Meier plots of survival of patients divided by low or high expression (below or above median) of hsa‐miR‐6069.
**Fig. S9.** Kaplan–Meier plots of survival of patients divided by low or high expression (below or above median) of hsa‐miR‐4745‐5p.
**Fig. S10.** Kaplan–Meier plots of survival of patients divided by low or high expression (below or above median) of hsa‐miR‐19b‐3p.
**Fig. S11.** Kaplan–Meier plots of survival of patients divided by pT of their tumor.
**Fig. S12.** PCA plots of mRNA and miRNA data (first two components), with annotation by disease stage, compared between the study cohort, the TCGA‐GA cohort, and the TCGA‐HS cohort.
**Fig. S13.** PCA plots of mRNA and miRNA data (first two components), with annotation by OS status, compared between the study cohort, the TCGA‐GA cohort, and the TCGA‐HS cohort.
**Fig. S14.** PCA plots of mRNA and miRNA data (first two components), with annotation by pathologic tumor size (pT), compared between the study cohort, the TCGA‐GA cohort, and the TCGA‐HS cohort.
**Fig. S15.** PCA plots of mRNA and miRNA data (first two components), with annotation by pathologic node status, compared between the study cohort, the TCGA‐GA cohort, and the TCGA‐HS cohort.
**Fig. S16.** PCA plots of mRNA and miRNA data (first two components), with annotation by progesterone receptor (PR) status, compared between the study cohort, the TCGA‐GA cohort, and the TCGA‐HS cohort.
**Fig. S17.** Results of supervised DIABLO modeling based on disease stage or OS status as discriminants, using the TCGA‐HS cohort—hierarchically clustered heatmap of features and samples.
**Fig. S18.** Results of supervised DIABLO modeling based on pathologic tumor size (pT) or node status (pN) as discriminants, using the TCGA‐HS cohort—hierarchically clustered heatmap of features and samples.
**Fig. S19.** Results of supervised DIABLO modeling based on PR status as the discriminant, using the TCGA‐HS cohort—hierarchically clustered heatmap of features and samples.

**Table S1.** Full list of oxysterol‐related genes studied by targeted DNA sequencing.
**Table S2.** Clinical characteristics of the patients.
**Table S3.** *multiMiR* databases used in the study.
**Table S4.** miRNA‐miRNA interactions with strong correlation (r ≥ 0.8) and FDR adj. p ≤ 0.05 in the original cohort, compared with values for the same interactions from validation analyses.
**Table S5.** Largest miRNA‐miRNA correlation coefficient (Spearman) differences between patients separated by the molecular subtype, menopause status, and progesterone receptor status.
**Table S6.** mRNA‐mRNA interactions with FDR adj. p ≤ 0.05 in the original cohort, compared with values for the same interactions from validation analyses (FDR adj. p ≤ 0.05).
**Table S7.** Largest mRNA‐mRNA correlation coefficient (Spearman) differences between patients separated by the molecular subtype, menopause status, and progesterone receptor status.
**Table S8.** mRNA‐miRNA interactions with FDR adj. p ≤ 0.05 in the original cohort, compared with values for the same interactions from validation analyses (FDR adj. p ≤ 0.05).
**Table S9.** Largest mRNA‐miRNA correlation coefficient (Spearman) differences between patients separated by the molecular subtype, menopause status, and progesterone receptor status.
**Table S10.** miRNAs whose expression above median associated with survival in the study cohort.
**Table S11.** Cohorts considered for DIABLO modeling.
**Table S12.** Minimal discriminatory mRNA‐miRNA signatures of the DIABLO models on the TCGA‐HS cohort.

## Data Availability

The miRNA data were processed according to the MIAME guidelines [59] and deposited in NCBI's Gene Expression Omnibus (GEO, [60]) repository under GEO: GSE225292. The RNA sequencing data were deposited under GEO: GSE225327 in accordance with the MINSEQE guidelines [61] and to NCBI's Sequence Read Archive (SRA) under SRA: PRJNA935263. DNA sequencing data aligned to the GRCh37 reference genome (BAM files) were deposited in SRA: PRJNA802324. The study is also available in GEO as a SuperSeries GEO: GSE225328. Any remaining data not part of the manuscript or supplemental files will be provided upon reasonable request to the corresponding author.
