# Supplementary material for: Integrative analysis of mRNA and miRNA expression profiles and somatic variants in oxysterol signaling in early‐stage luminal breast cancer
Source: Mol Oncol. 2023 Aug 18;17(10):2074–89. doi: 10.1002/1878-0261.13495 (PMC10552891; doi:10.1002/1878-0261.13495)
Supplement: Supplementary file 1 — Fig. S1. Additional groups of co‐expressed miRNAs. Fig. S2. Groups of co‐expressed miRNAs that were mostly not confirmed by TCGA validation. Fig. S3. Clustered heatmap of mRNA‐mRNA correlations across all 113 oxysterol‐related genes. Fig. S4. Volcano plots of differentially expressed genes. Fig. S5. Kaplan–Meier plots of survival of patients divided by low or high expression (below or above median) of LDLR mRNA. Fig. S6. Kaplan–Meier plots of survival of patients divided by low or high expression (below or above median) of PPARGC1A mRNA. Fig. S7. Kaplan–Meier plots of survival of patients divided by zero or nonzero expression (none or any level of expression detected) of CYP3A4 mRNA. Fig. S8. Kaplan–Meier plots of survival of patients divided by low or high expression (below or above median) of hsa‐miR‐6069. Fig. S9. Kaplan–Meier plots of survival of patients divided by low or high expression (below or above median) of hsa‐miR‐4745‐5p. Fig. S10. Kaplan–Meier plots of survival of patients divided by low or high expression (below or above median) of hsa‐miR‐19b‐3p. Fig. S11. Kaplan–Meier plots of survival of patients divided by pT of their tumor. Fig. S12. PCA plots of mRNA and miRNA data (first two components), with annotation by disease stage, compared between the study cohort, the TCGA‐GA cohort, and the TCGA‐HS cohort. Fig. S13. PCA plots of mRNA and miRNA data (first two components), with annotation by OS status, compared between the study cohort, the TCGA‐GA cohort, and the TCGA‐HS cohort. Fig. S14. PCA plots of mRNA and miRNA data (first two components), with annotation by pathologic tumor size (pT), compared between the study cohort, the TCGA‐GA cohort, and the TCGA‐HS cohort. Fig. S15. PCA plots of mRNA and miRNA data (first two components), with annotation by pathologic node status, compared between the study cohort, the TCGA‐GA cohort, and the TCGA‐HS cohort. Fig. S16. PCA plots of mRNA and miRNA data (first two components), with annotation by progesterone [file MOL2-17-2074-s002.pdf]

Integrative analysis of mRNA-miRNA expression profiles and somatic variants in oxysterol signaling in early-stage luminal breast cancer

Petr Holý[1,2,3], Veronika Brynychová[1,3], Karolína Šeborová[1,3], Vojtěch Haničinec[3], Renata Koževnikovová[4], Markéta Trnková[5], David Vrána[6], Jiří Gatěk[7], Kateřina Kopečková[8], Marcela Mrhalová[9], and Pavel Souček[1,3]*

Supplementary Figures

Table of contents:

Figure	Title
**S1**	Additional groups of co-expressed miRNAs
**S2**	Groups of co-expressed miRNAs that were mostly not confimed by TCGA validation
**S3**	Clustered heatmap of mRNA-mRNA correlations across all 113 oxysterol-related genes
**S4**	Volcano plots of differentially expressed genes
**S5**	Kaplan-Meier plots of survival of patients divided by low or high expression (below or above median) of *LDLR* mRNA
**S6**	Kaplan-Meier plots of survival of patients divided by low or high expression (below or above median) of *PPARGC1A* mRNA
**S7**	Kaplan-Meier plots of survival of patients divided by zero or non-zero expression (none or any level of expression detected) of *CYP3A4* mRNA
**S8**	Kaplan-Meier plots of survival of patients divided by low or high expression (below or above median) of hsa-miR-6069
**S9**	Kaplan-Meier plots of survival of patients divided by low or high expression (below or above median) of hsa-miR-4745-5p
**S10**	Kaplan-Meier plots of survival of patients divided by low or high expression (below or above median) of hsa-miR-19b-3p
**S11**	Kaplan-Meier plots of survival of patients divided by pT of their tumor
**S12**	PCA plots of mRNA and miRNA data (first two components), with annotation by disease stage, compared between the study cohort, the TCGA-GA cohort, and the TCGA-HS cohort
**S13**	PCA plots of mRNA and miRNA data (first two components), with annotation by OS status, compared between the study cohort, the TCGA-GA cohort, and the TCGA-HS cohort
**S14**	PCA plots of mRNA and miRNA data (first two components), with annotation by pathologic tumor size (pT), compared between the study cohort, the TCGA-GA cohort, and the TCGA-HS cohort
**S15**	PCA plots of mRNA and miRNA data (first two components), with annotation by pathologic node status, compared between the study cohort, the TCGA-GA cohort, and the TCGA-HS cohort

**S16**	PCA plots of mRNA and miRNA data (first two components), with annotation by progesterone receptor (PR) status, compared between the study cohort, the TCGA-GA cohort, and the TCGA-HS cohort
**S17**	Results of supervised DIABLO modeling based on disease stage or OS status as discriminants, using the TCGA-HS cohort – hierarchically clustered heatmap of features and samples
**S18**	Results of supervised DIABLO modeling based on pathologic tumor size (pT) or node status (pN) as discriminants, using the TCGA-HS cohort – hierarchically clustered heatmap of features and samples
**S19**	Results of supervised DIABLO modeling based on PR status as the discriminant, using the TCGA-HS cohort – hierarchically clustered heatmap of features and samples

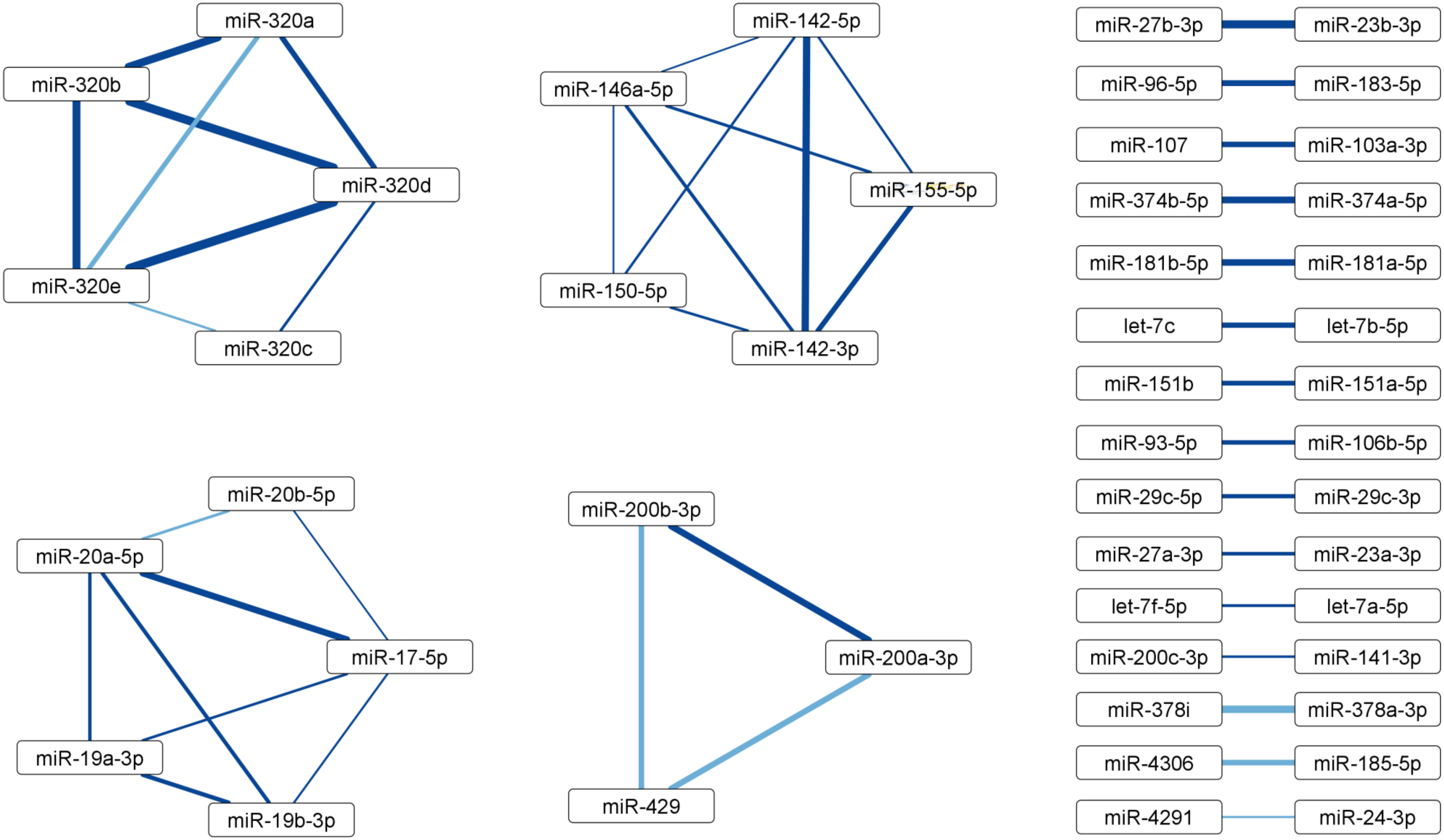

Figure S1 – Additional groups of co-expressed miRNAs. *Line thickness corresponds to strength of correlation (r between 0.80 and 0.99). Interaction confirmed either in both TCGA datasets (dark blue), or just one (light blue). "hsa-" omitted from miRNA names for brevity.*

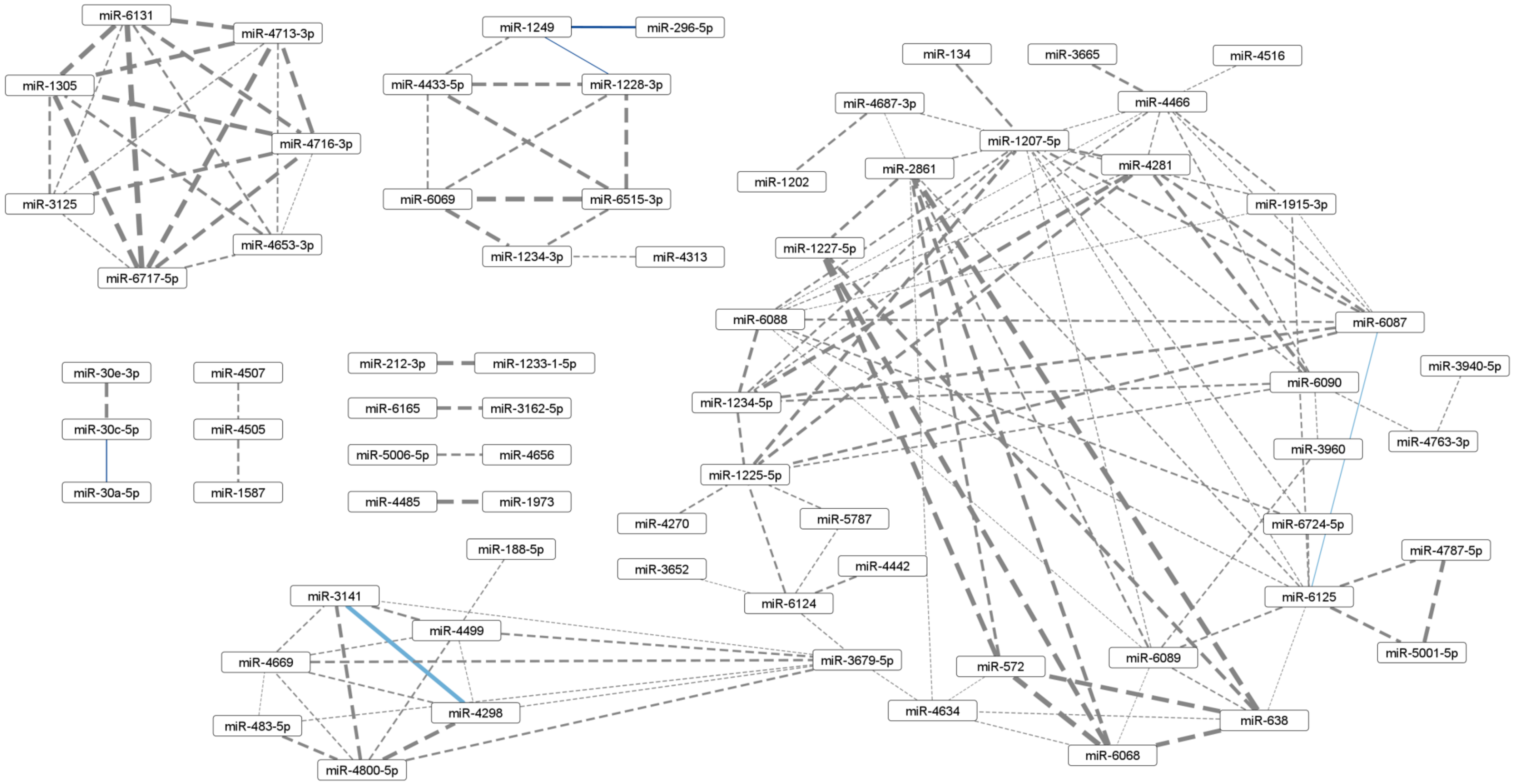

Figure S2 – Groups of co-expressed miRNAs that were mostly not confimed by TCGA validation. *Line thickness corresponds to strength of correlation (r between 0.80 and 0.99). Grey dashed lines – found in study cohort but not in TCGA. Some interactions were confirmed either in both TCGA datasets (dark blue solid lines), or just one (light blue solid lines). "hsa-" omitted from miRNA names for brevity.*

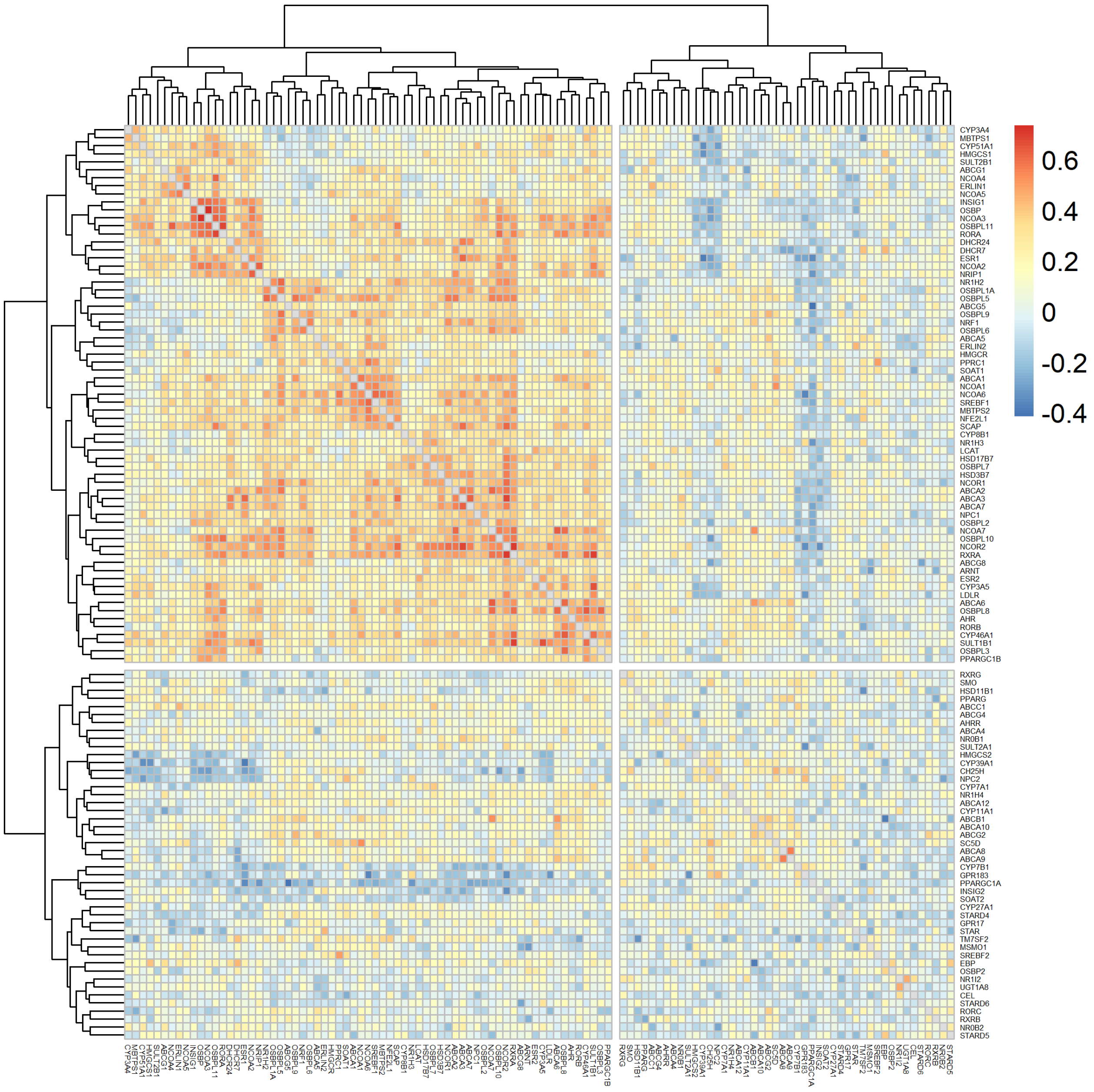

Figure S3 – Clustered heatmap of mRNA-mRNA correlations across all 113 oxysterol-related genes. *Color coding represents Spearman correlation coefficient (r). For clarity, all instances of r=1 greyed out.*

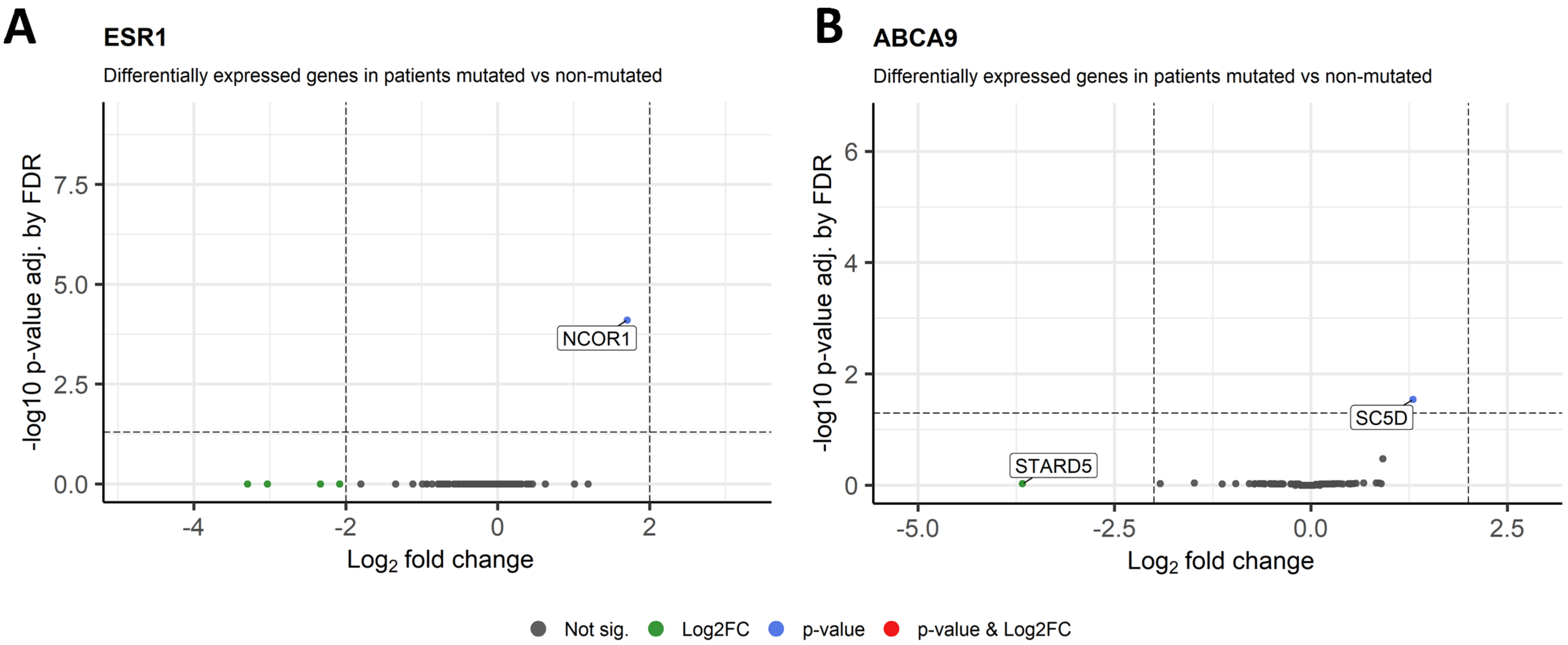

Figure S4 – Volcano plots of differentially expressed genes. *Patient groups separated by mutation status of (**A**) ESR1, (**B**) ABCA9. Vertical dashed lines show threshold for fold change (log2FC = ±2), horizontal dashed lines show threshold for significant p-value after FDR (0.05).*

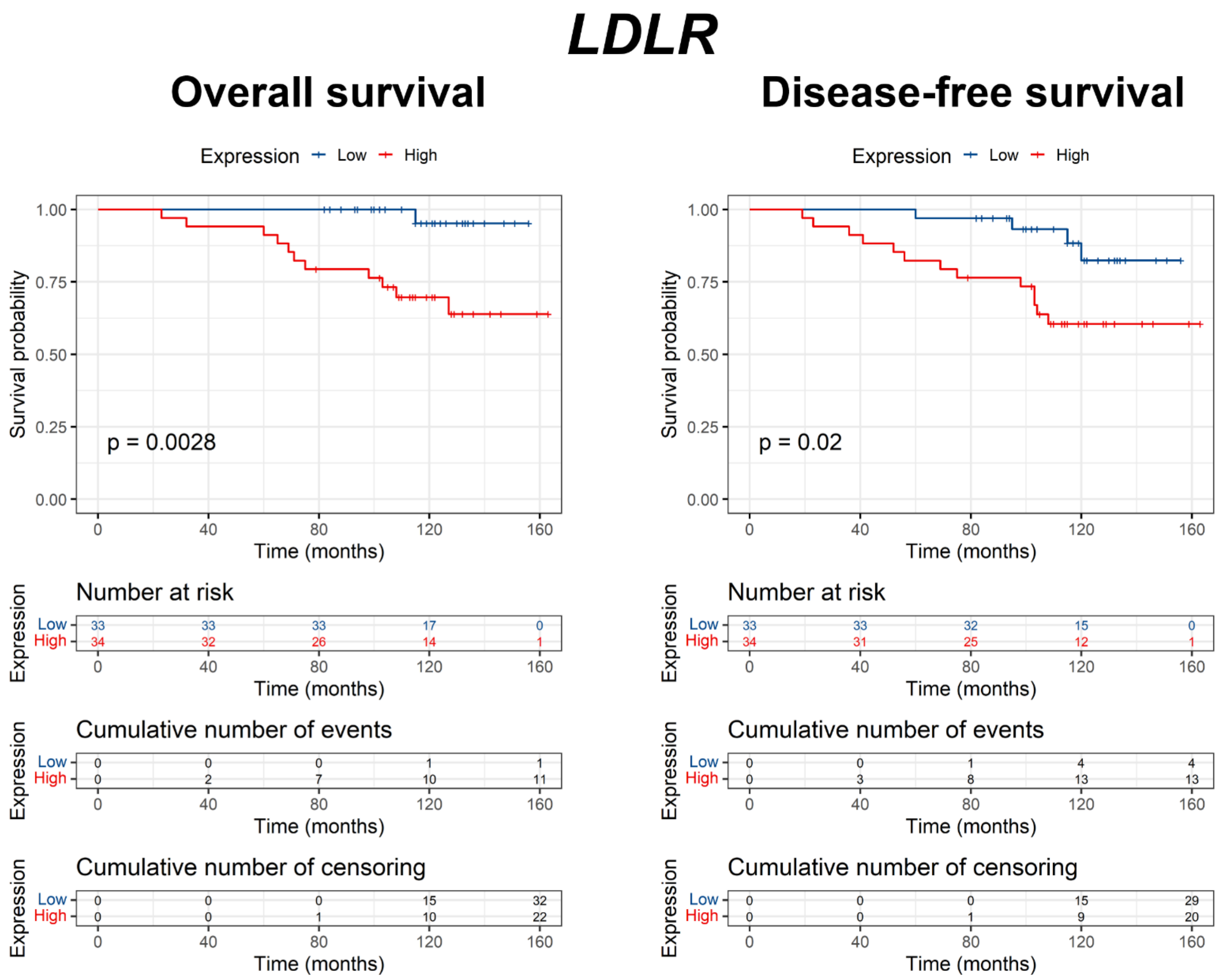

Figure S5 – Kaplan-Meier plots of survival of patients divided by low or high expression (below or above median) of *LDLR* mRNA.

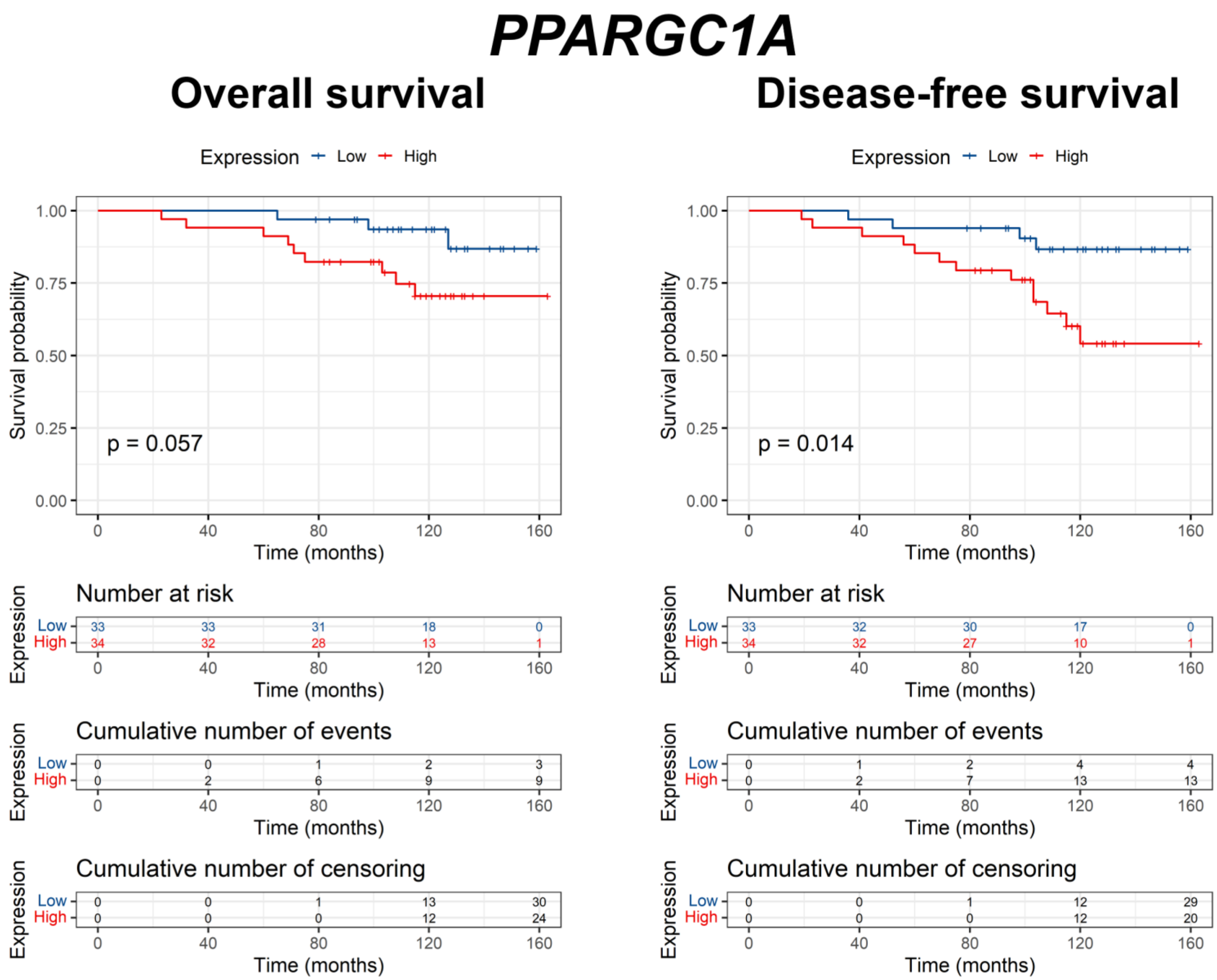

Figure S6 – Kaplan-Meier plots of survival of patients divided by low or high expression (below or above median) of *PPARGC1A* mRNA.

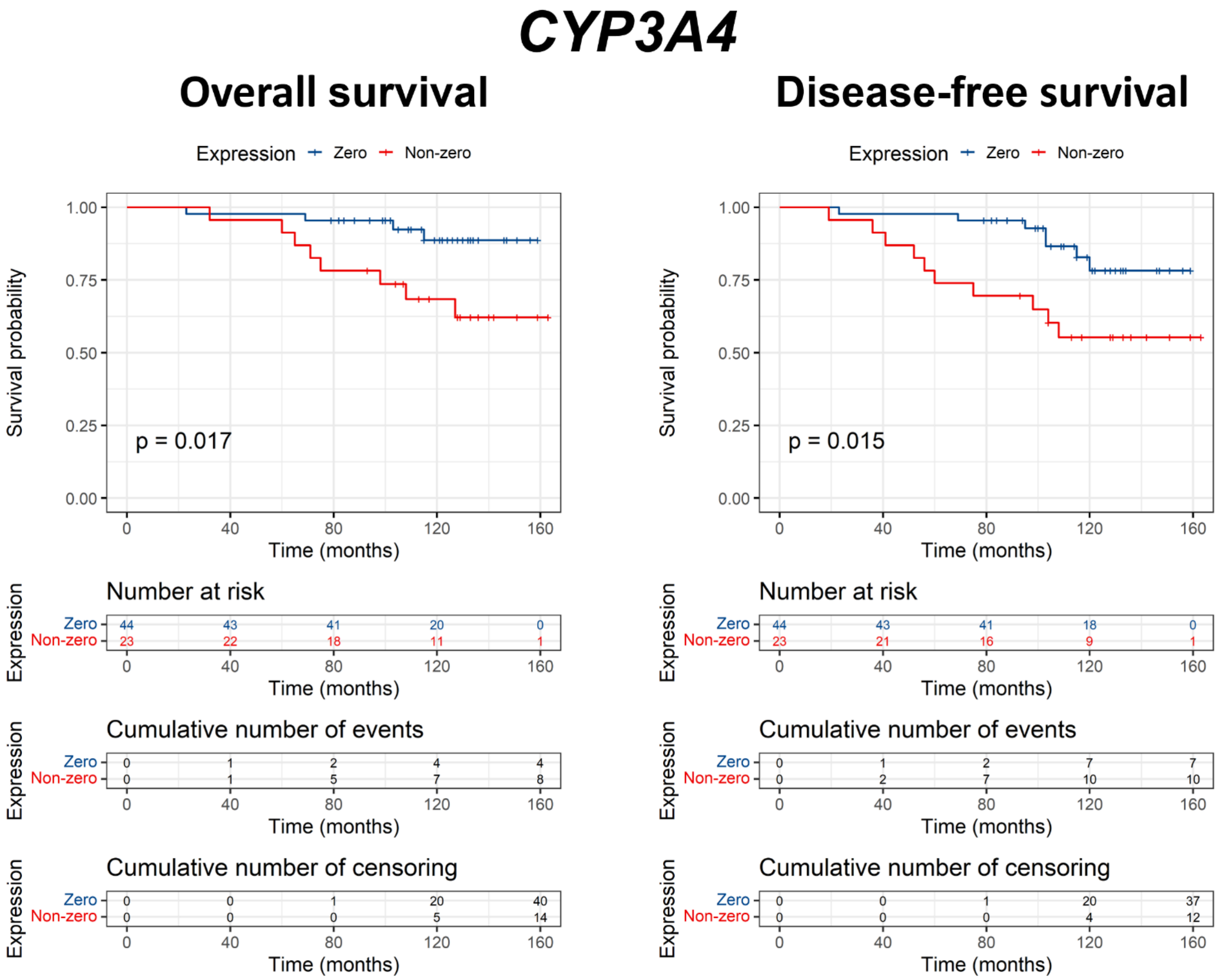

Figure S7 – Kaplan-Meier plots of survival of patients divided by zero or non-zero expression (none or any level of expression detected) of *CYP3A4* mRNA.

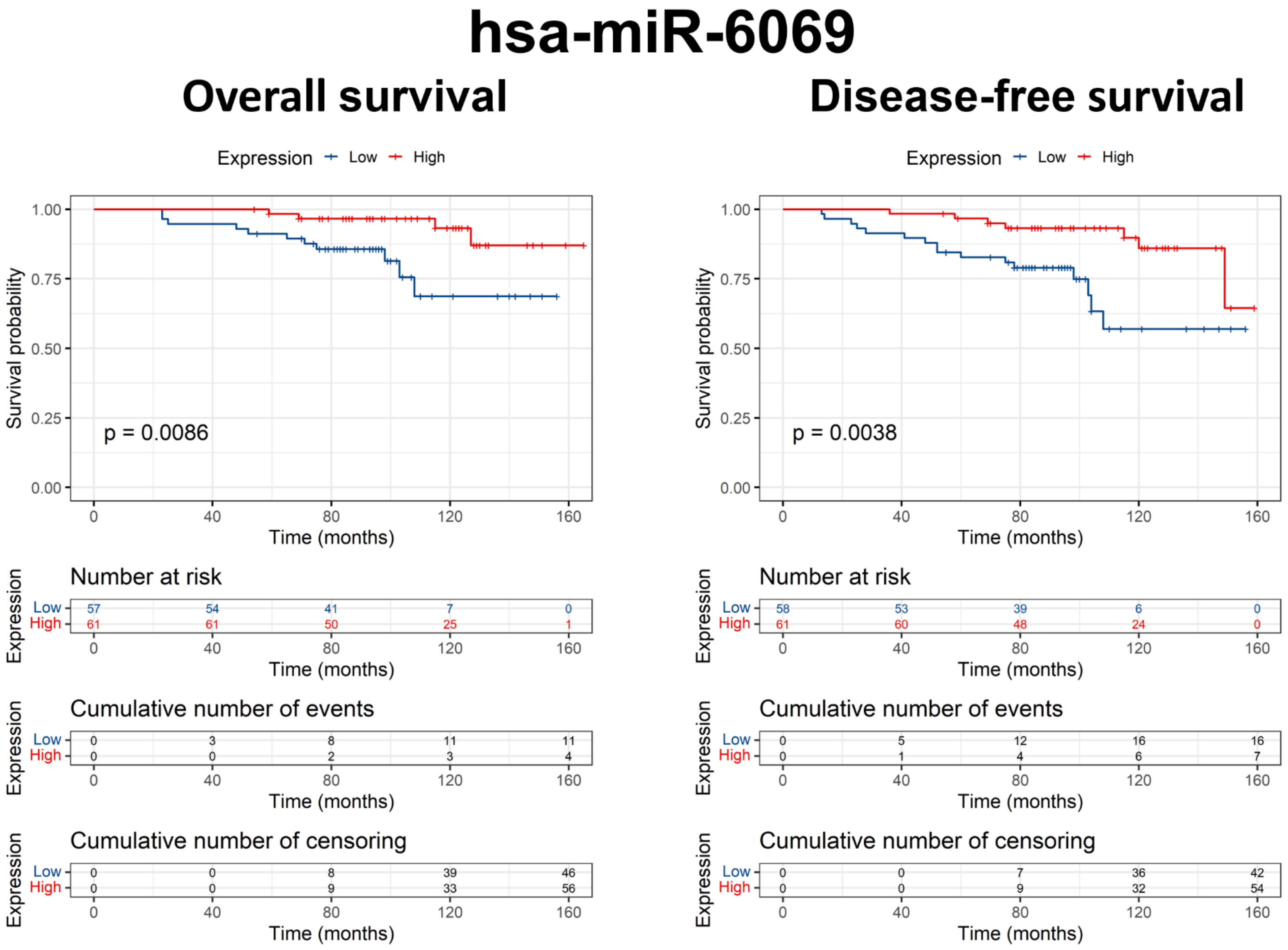

Figure S8 – Kaplan-Meier plots of survival of patients divided by low or high expression (below or above median) of hsa-miR-6069.

hsa-miR-4745-5p

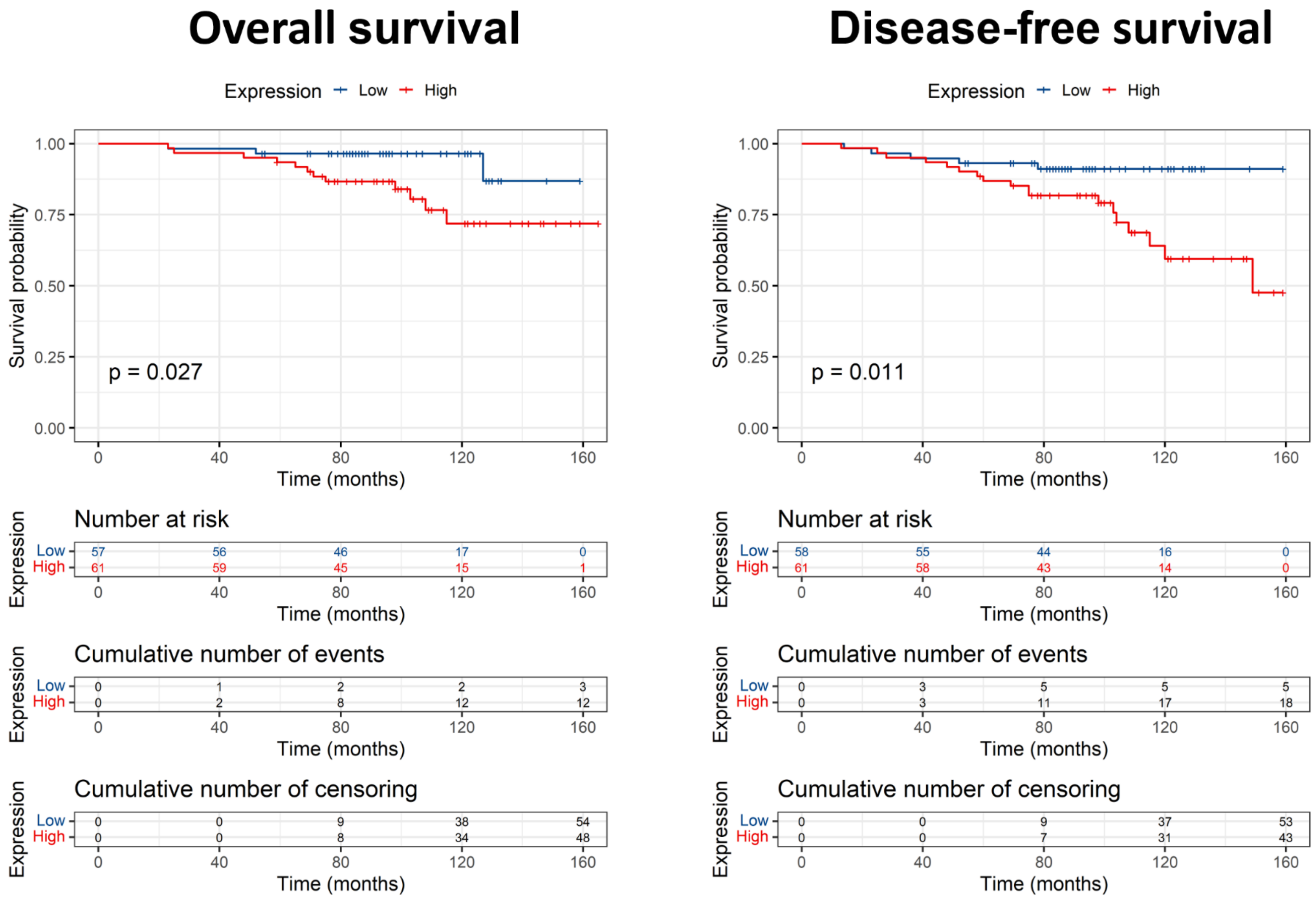

Figure S9 – Kaplan-Meier plots of survival of patients divided by low or high expression (below or above median) of hsa-miR-4745-5p.

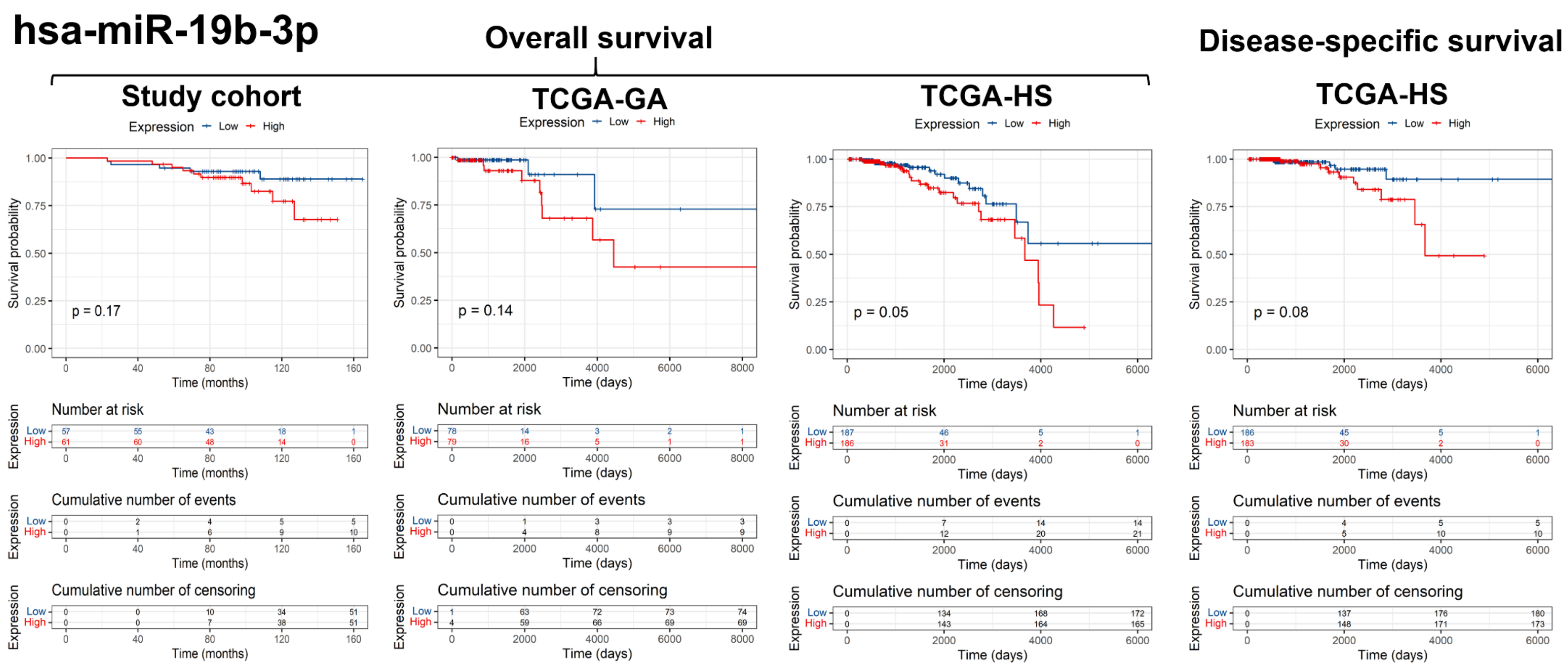

Figure S10 – Kaplan-Meier plots of survival of patients divided by low or high expression (below or above median) of hsa-miR-19b-3p.

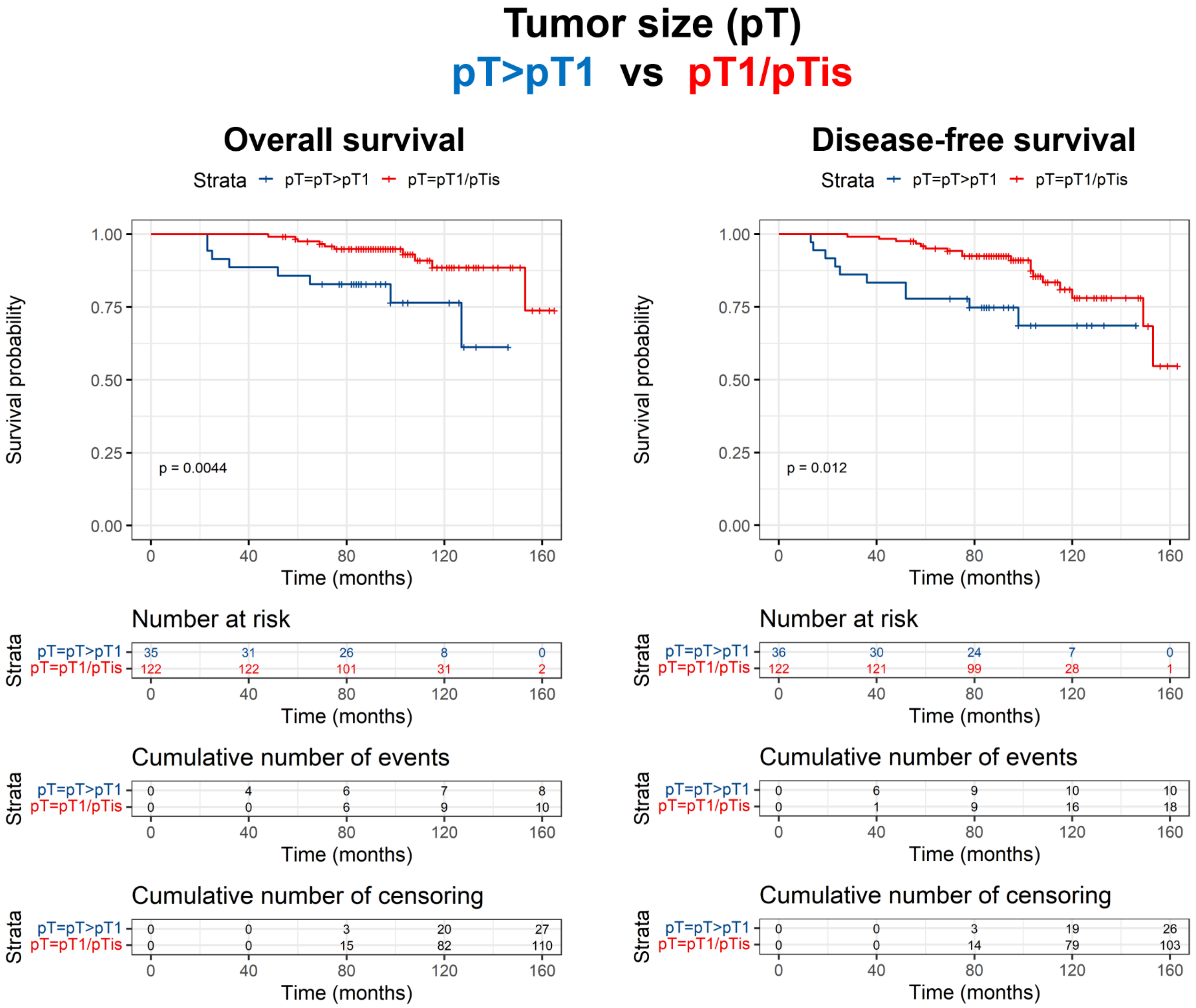

Figure S11 – Kaplan-Meier plots of survival of patients divided by pT of their tumor.

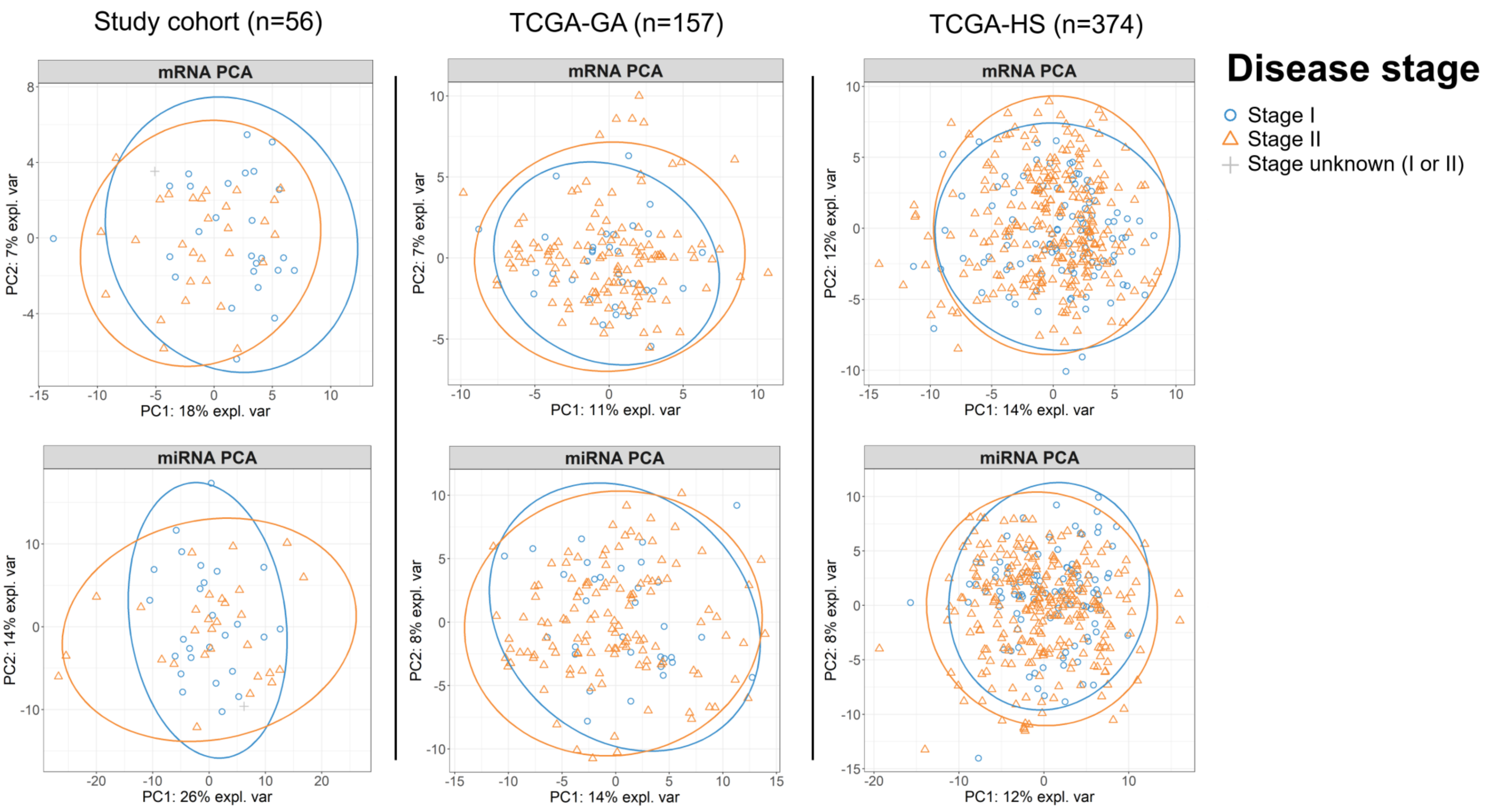

Figure S12 – PCA plots of mRNA and miRNA data (first two components), with annotation by disease stage, compared between the study cohort, the TCGA-GA cohort, and the TCGA-HS cohort.

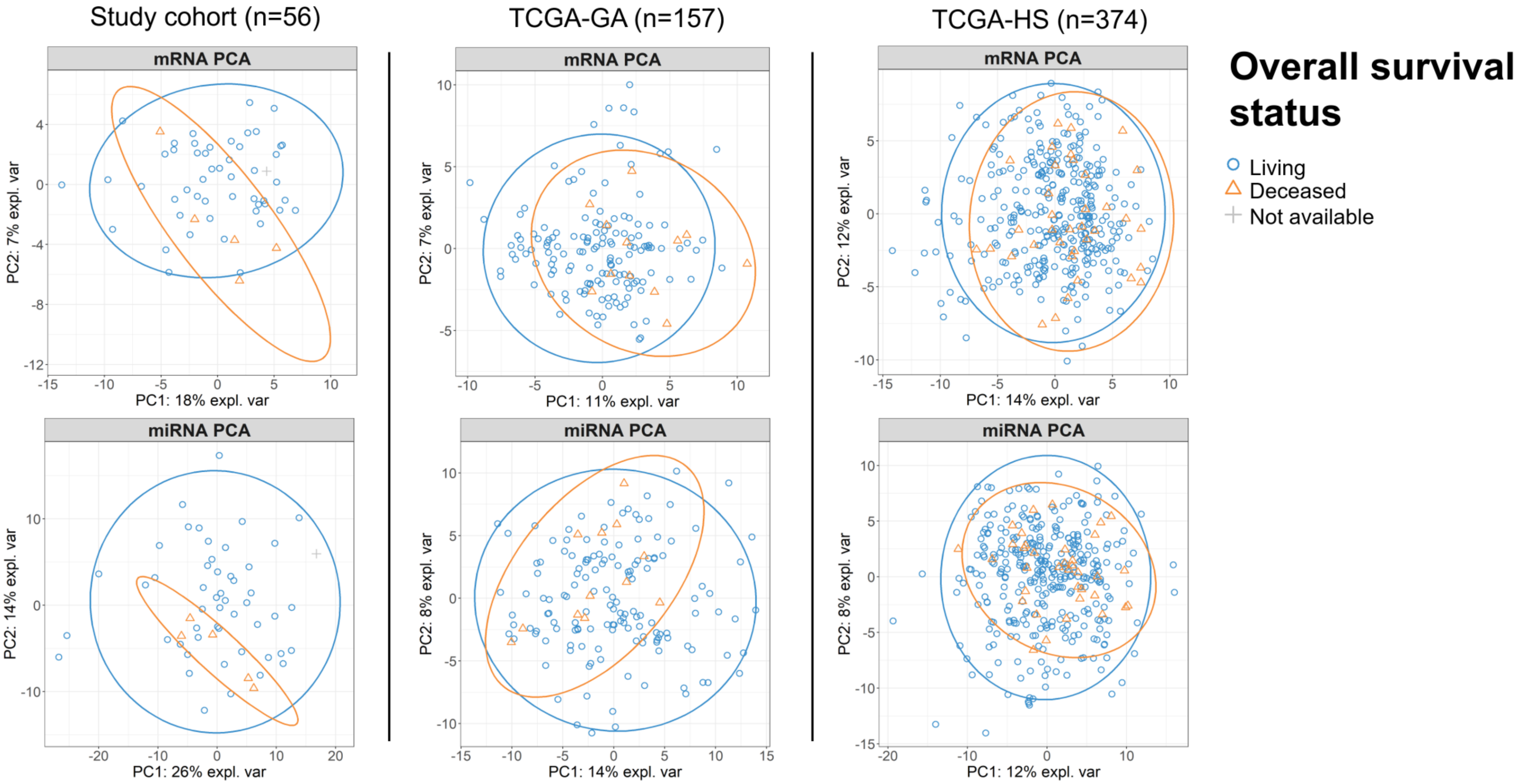

Figure S13 – PCA plots of mRNA and miRNA data (first two components), with annotation by OS status, compared between the study cohort, the TCGA-GA cohort, and the TCGA-HS cohort.

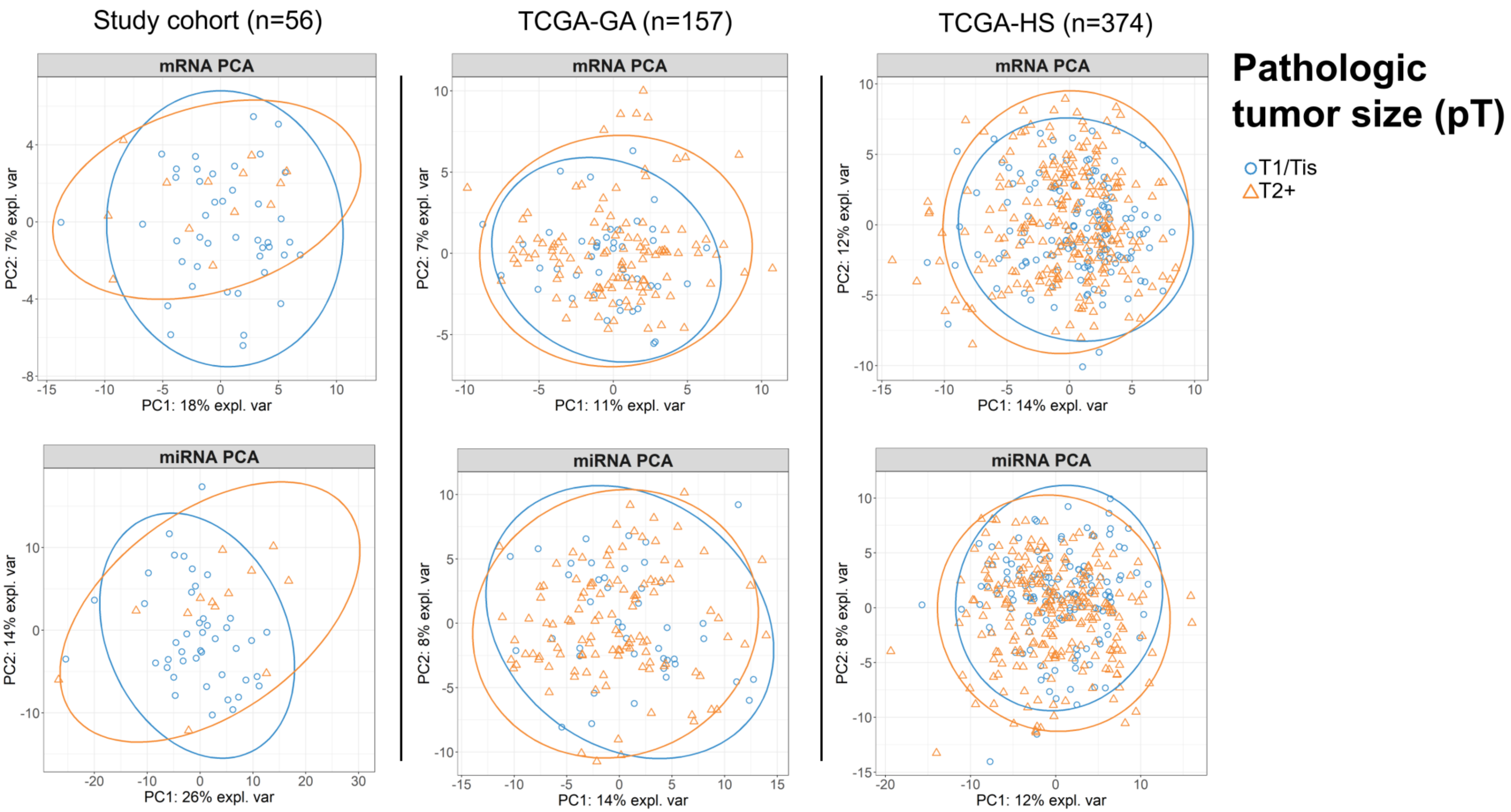

Figure S14 – PCA plots of mRNA and miRNA data (first two components), with annotation by pathologic tumor size (pT), compared between the study cohort, the TCGA-GA cohort, and the TCGA-HS cohort. *Tis = tumor in situ*.

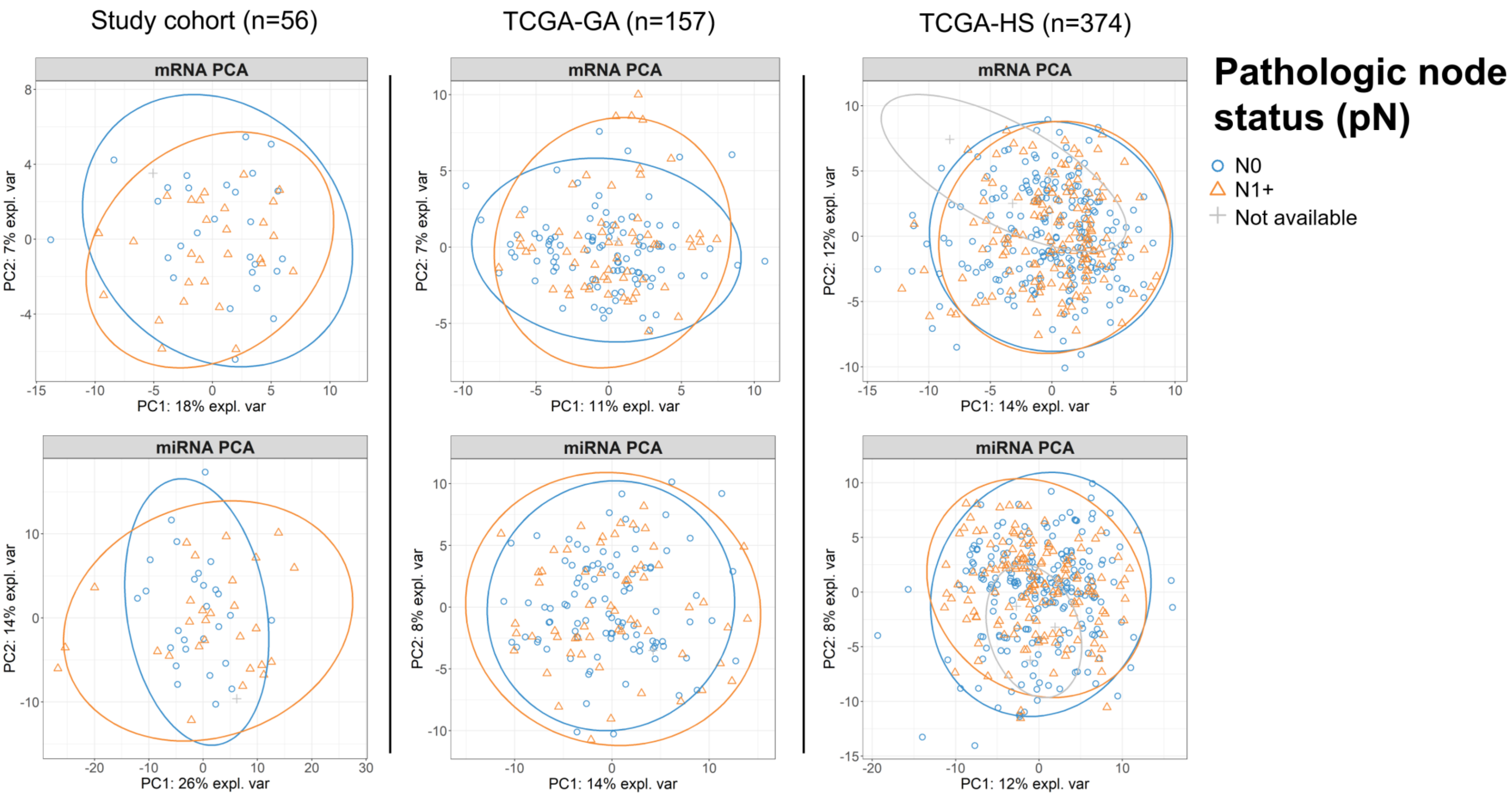

Figure S15 – PCA plots of mRNA and miRNA data (first two components), with annotation by pathologic node status, compared between the study cohort, the TCGA-GA cohort, and the TCGA-HS cohort.

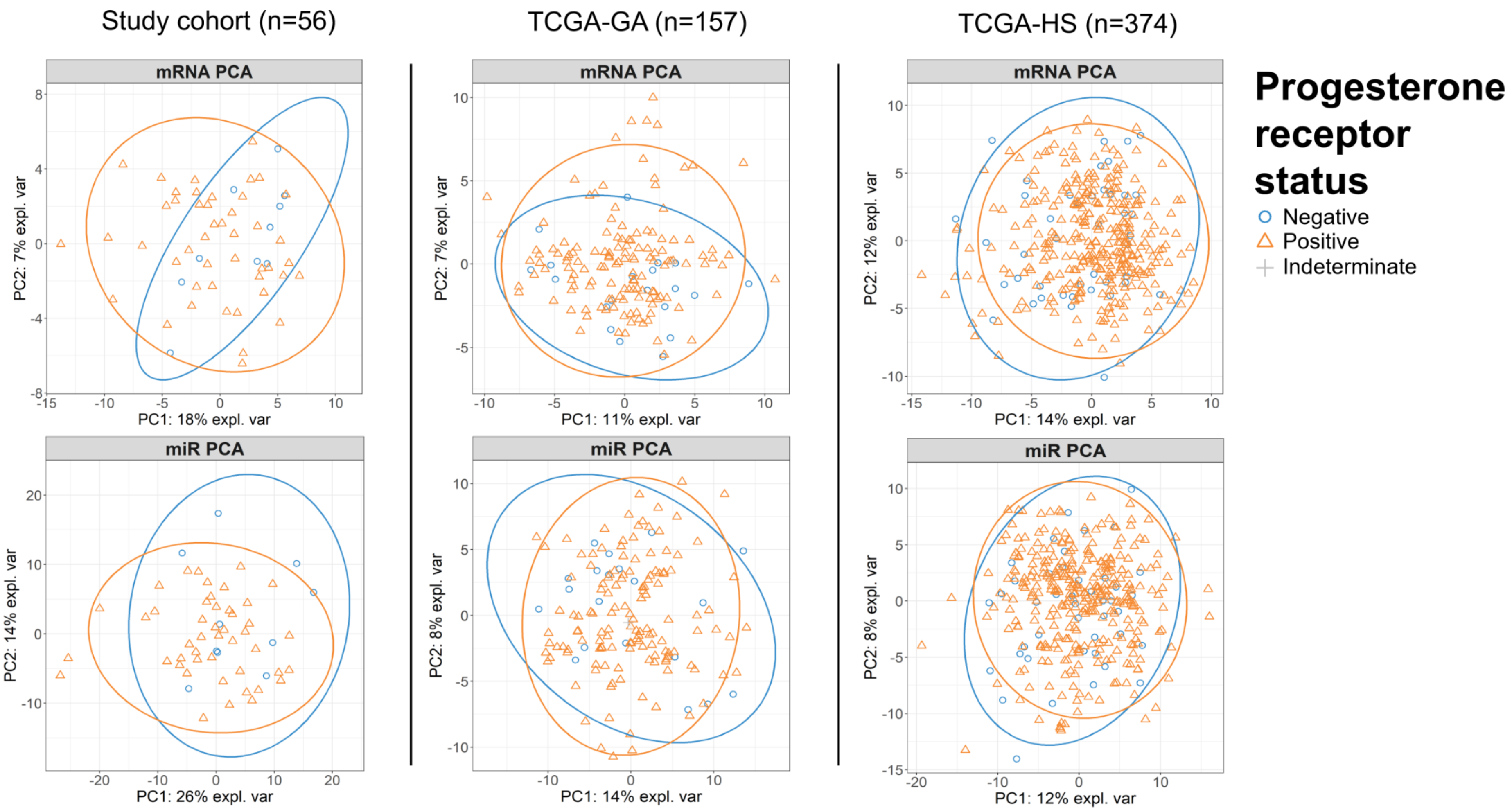

Figure S16 – PCA plots of mRNA and miRNA data (first two components), with annotation by progesterone receptor (PR) status, compared between the study cohort, the TCGA-GA cohort, and the TCGA-HS cohort.

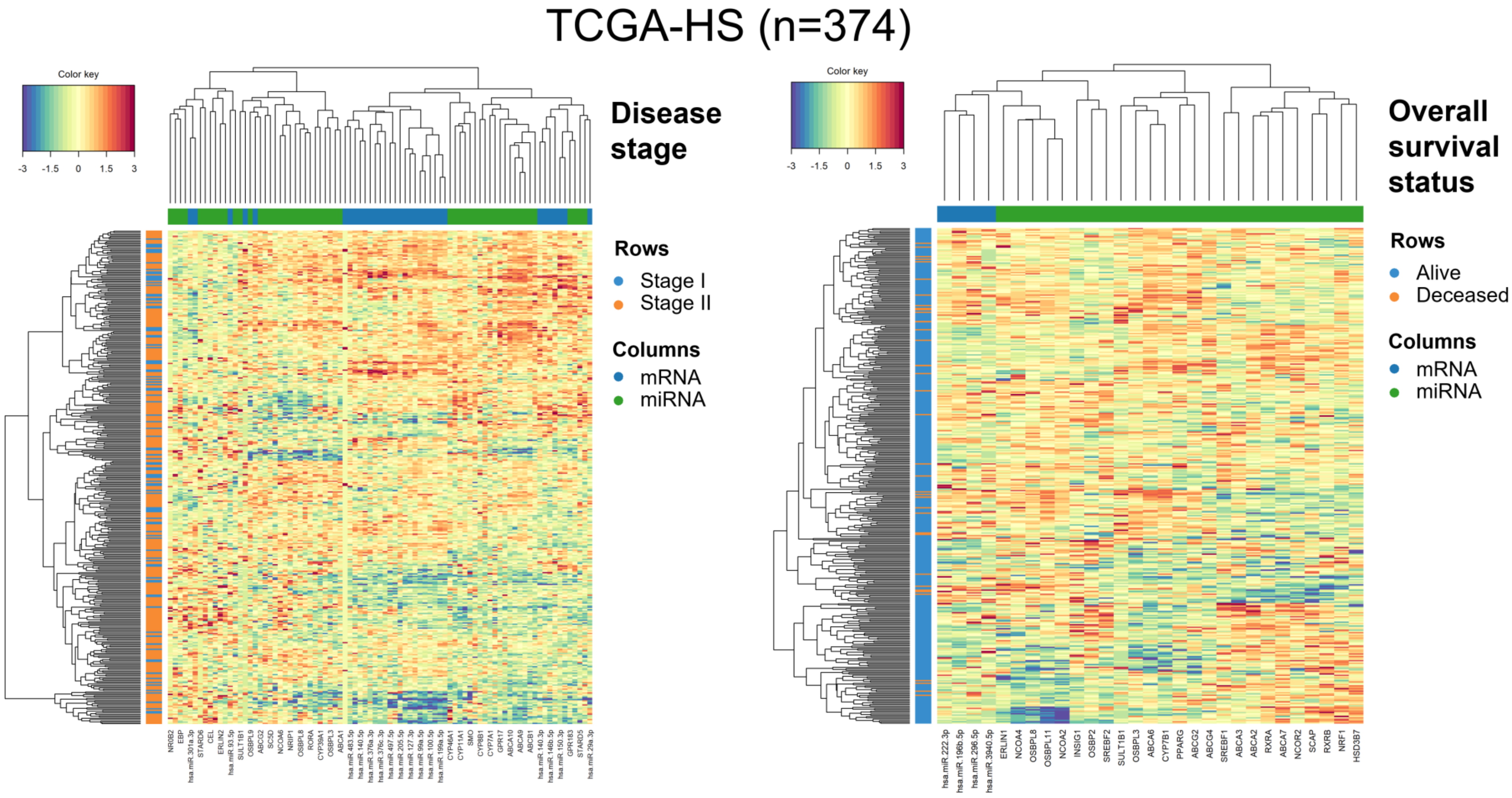

Figure S17 – Results of supervised DIABLO modeling based on disease stage or OS status as discriminants, using the TCGA-HS cohort – hierarchically clustered heatmap of features and samples. *No clustering between samples is apparent, indicating that the model is a poor predictor.*

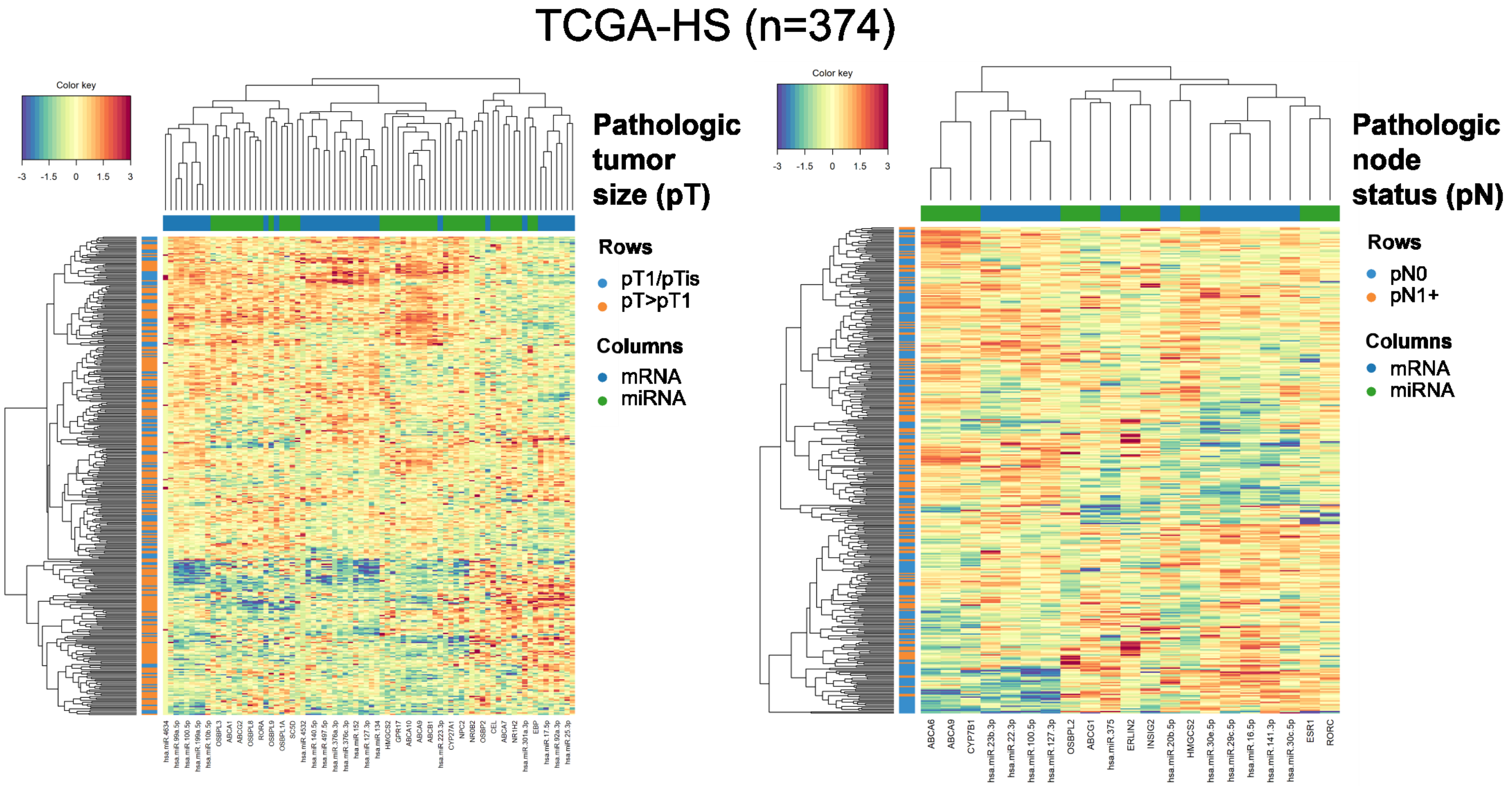

Figure S18 – Results of supervised DIABLO modeling based on pathologic tumor size (pT) or node status (pN) as discriminants, using the TCGA-HS cohort – hierarchically clustered heatmap of features and samples. *The signature (Table S8) appears weakly associated with high pT, however, the model is still a poor predictor. pTis = tumor in situ*

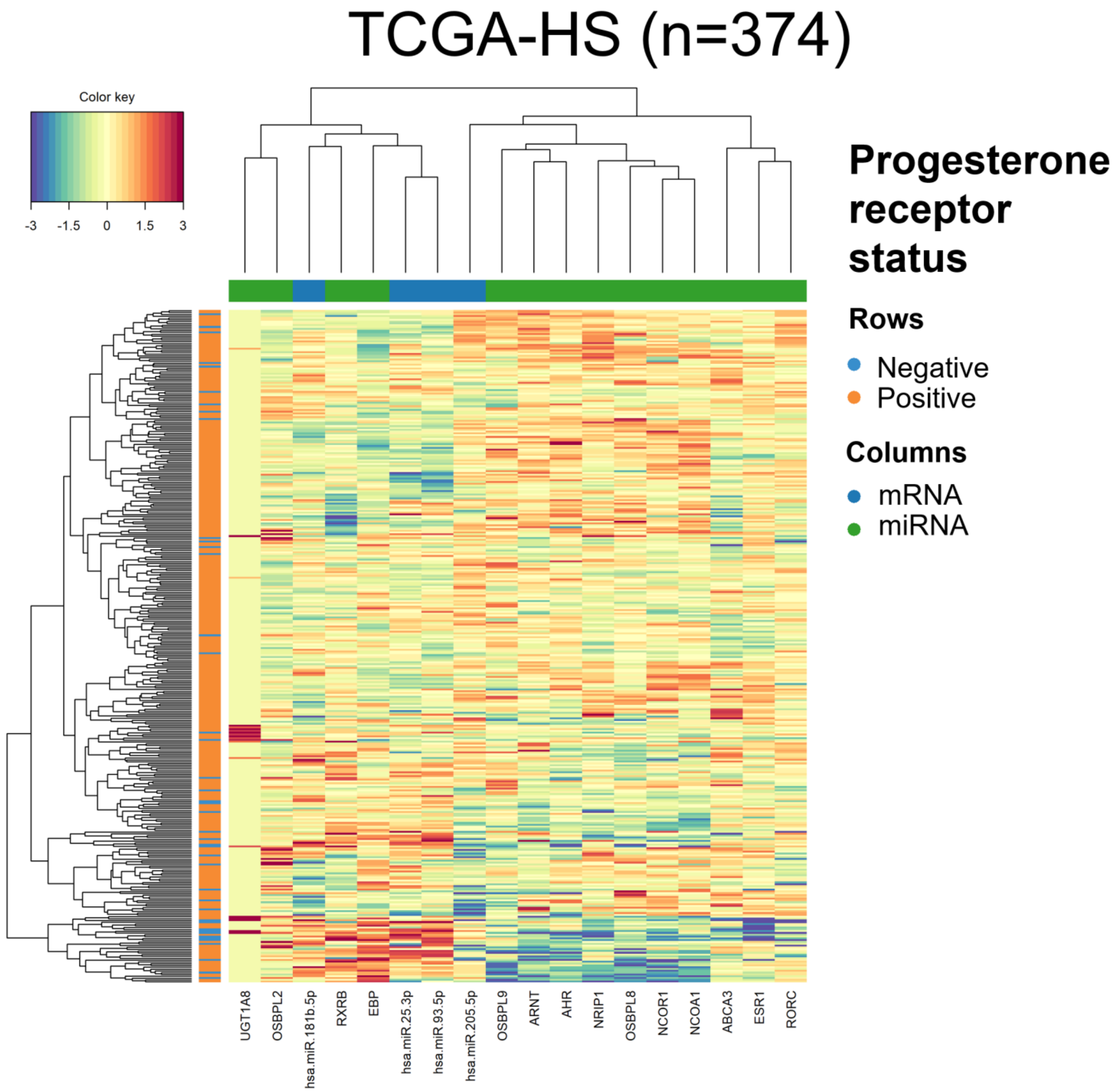

Figure S19 – Results of supervised DIABLO modeling based on PR status as the discriminant, using the TCGA-HS cohort – hierarchically clustered heatmap of features and samples. *The signature (Table S8) appears weakly associated with negative PR status, however, the model is still a poor predictor and more data would be needed.*
